# Functional differences between CLL‐ and ALL‐derived CAR T cells in a 3D tumor microenvironment highlight CXCR4 and IL‐10 as potential modulatory targets

**DOI:** 10.1002/hem3.70279

**Published:** 2025-12-08

**Authors:** Janin Dingfelder, Michael Aigner, Jana Lindacher, Pascal Lukas, Cindy Flamann, Gina Nusser, Katharina Zimmermann, Axel Schambach, Frederik Graw, Simon Völkl, Heiko Bruns, Manuela Krumbholz, Martina Haibach, Jochen Wilke, Andreas Mackensen, Gloria Lutzny‐Geier

**Affiliations:** ^1^ Department of Internal Medicine 5, Hematology and Oncology Universitätsklinikum Erlangen Friedrich‐Alexander‐Universität Erlangen‐Nürnberg (FAU) Erlangen Germany; ^2^ Bavarian Cancer Research Center (BZKF) Erlangen Germany; ^3^ Deutsches Zentrum für Immuntherapie (DZI) Friedrich‐Alexander‐Universität Erlangen‐Nürnberg Erlangen Germany; ^4^ Hannover Medical School (MHH), Institute of Experimental Hematology Hannover Germany; ^5^ Pediatric Oncology and Hematology, Department of Pediatrics and Adolescent Medicine University Hospital Erlangen Erlangen Germany; ^6^ Practice for Internal Medicine, Oncology and Haematology Erlangen Germany; ^7^ Practice for Oncology and Haematology Fürth Germany

## Abstract

Despite advances in targeted therapies, treatment of chronic lymphocytic leukemia (CLL) remains challenging, highlighting the urgent need for effective new therapeutic strategies. Although chimeric antigen receptor (CAR) T‐cell therapy dramatically improved outcomes in acute lymphoblastic leukemia (ALL), its efficacy in CLL is limited. We hypothesize that this disparity results from pronounced CAR T‐cell exhaustion and the immunosuppressive tumor microenvironment (TME) in CLL. We utilized an autologous 3D TME co‐culture model to investigate the functionality of CAR T cells derived from CLL and ALL patients within physiologically relevant conditions. Our results revealed increased exhaustion levels and diminished cytotoxicity of CAR T cells from CLL patients compared to those from ALL patients. Importantly, combining CAR T‐cell treatment with interleukin‐10 (IL‐10) or CXCR4 blockade effectively improved cytotoxicity against CLL cells, even in stromal‐protected regions within the 3D model. These findings offer insights into CAR T‐cell dysfunction in CLL and support novel TME‐targeted combination strategies to improve clinical outcomes.

## Graphical abstract

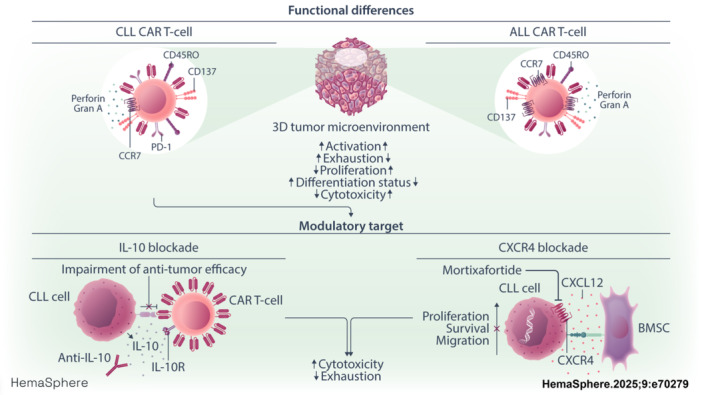

## INTRODUCTION

Chronic lymphocytic leukemia (CLL) represents the most common leukemia among adults in Western countries, characterized by the accumulation of mature, monoclonal B lymphocytes in peripheral blood, bone marrow, and lymphoid tissues.^1^ Despite significant advances in targeted therapies, including B‐cell receptor (BCR) signaling inhibitors and BCL–2 antagonists, CLL remains incurable for most patients, highlighting the need for novel therapeutic strategies.^2^, ^3^, ^4^ Over the past decade, chimeric antigen receptor (CAR) T‐cell immunotherapy has become a promising option for high‐risk relapsed/refractory (r/r) CLL patients with resistance to standard therapies. While CAR T‐cell therapy has revolutionized treatment outcomes for patients with aggressive B‐cell malignancies such as acute lymphoblastic leukemia (ALL), response rates in CLL remain limited, with complete remissions achieved in only 18%–29% of patients, indicating fundamental differences in disease biology and immune response modulation.^5^, ^6^, ^7^, ^8^

Recent studies indicate that the reduced therapeutic efficacy of CAR T cells in CLL is largely due to disease‐specific T‐cell alterations and a more suppressive and protective tumor microenvironment (TME). Constant antigen‐stimulation drives chronic activation, impaired synapse formation, elevated effector differentiation, and premature exhaustion of CAR T cells, while extrinsic signals from the TME further facilitate malignant cell survival and immune evasion.^9^, ^10^, ^11^, ^12^ Particularly, the interplay between CAR T cells, malignant B cells, and stromal cells within protective niches appears to influence therapeutic outcomes. Conventional two‐dimensional (2D) in vitro cultures often fail to fully mimic these complex interactions, limiting their predictive power regarding clinical efficacy.^13^, ^14^, ^15^, ^16^ To overcome these limitations, we established an innovative autologous three‐dimensional (3D) scaffold‐based co‐culture model that replicates essential features of the stromal bone marrow TME, including direct cell–cell interactions between malignant B cells, CAR T cells, and bone marrow–derived stromal cells (BMSCs). Utilizing this advanced 3D model, we investigated the hypothesis that CLL‐derived CAR T cells exhibit increased exhaustion and reduced cytotoxic function compared to ALL‐derived CAR T cells due to distinct disease‐specific immune interactions within the TME.

Among various immunosuppressive mechanisms described in CLL, interleukin‐10 (IL‐10) represents one of several factors that may contribute to immune dysfunction. Similar to B cells, CLL cells constitutively produce IL‐10 dependent on BCR signaling.^17^ Elevated serum IL‐10 levels in CLL patients have been associated with poor prognosis.^18^, ^19^ Within the TME, sustained IL‐10 secretion potentially contributes to functional impairment of CAR T cells, thus further reducing therapeutic efficacy. Recent findings indicate that IL‐10 production by CLL cells is regulated via the CXCL12–CXCR4–STAT3 signaling axis.^20^ Beyond high serum IL‐10 levels, CXCR4 overexpression correlates with poor prognosis and inferior therapy response in CLL.^21^, ^22^ CXCR4 plays a central role in mediating the interaction between CLL cells and the protective microenvironment through binding of CXCL12, which is abundantly secreted by stromal cells. This axis not only promotes CLL cell retention in protective niches but also enhances their survival and therapy resistance.^23^, ^24^, ^25^ Consequently, the CXCR4/CXCL12 signaling pathway may impair CAR T‐cell infiltration and suppress effector function within stromal‐supported regions, thereby limiting therapeutic efficacy in CLL. Consistent with this, previous bulk RNA sequencing analysis from a similar CLL‐derived 3D model lacking CAR T cells showed increased IL‐10 and CXCR4 expression in CLL cells located in protected stromal niches, reinforcing their potential role in the TME.^26^ Therefore, we hypothesized that IL‐10 neutralization or CXCR4 blockade could improve CAR T‐cell efficacy.

Our findings demonstrate substantial differences in the functional characteristics of CAR T cells derived from CLL versus ALL patients. Specifically, CLL‐derived CAR T cells showed significantly higher levels of exhaustion markers, diminished proliferative capacity, and reduced cytotoxic activity. Moreover, targeting IL‐10 or CXCR4 alongside CAR T‐cell therapy enhanced cytotoxicity in CLL, suggesting a promising strategy to overcome treatment resistance. Thus, this study not only provides insights into the mechanisms underlying CAR T‐cell dysfunction in CLL but also supports the development of combination therapies targeting the TME, potentially leading to improved clinical outcomes for patients with CLL.

## RESULTS

### Healthy donor‐derived CAR T cells are highly functional in 2D co‐culture

Before generating CAR T cells from patient‐derived peripheral blood mononuclear cells (PBMCs), transduction was initially performed using T cells from five healthy donors (HDs). The functional activity of the in‐house generated CAR T cells was confirmed through various functionality assays in 2D co‐culture with different target cells. CD4^+^ and CD8^+^ T cells were transduced with a lentiviral vector to express a third‐generation anti‐CD19 CAR (Figure S5A). As control, T cells underwent mock‐transduction using the same viral system without the CAR construct. T cells from all five HDs were efficiently transduced, showing similar levels of CD19 CAR expression (Figure S5B).

To determine target‐induced activation of T cells, mock‐transduced T cells or CAR T cells were co‐cultured with CD19^–^ U937 cell line as a negative control, autologous B cells, the CLL‐derived CD19^+^ Mec‐1 cell line, or the ALL‐derived CD19^+^ Nalm‐6 cell line at an effector‐to‐target (E:T) ratio of 1:1. CAR T cells showed CAR‐mediated activation upon target cell contact, evidenced by co‐expression of the early activation marker CD69^+^ and the specific activation marker CD137^+^ (Figure S5C).

To assess the cytotoxic capacity of CAR T cells, we performed a degranulation assay. Mock‐transduced T cells or CAR T cells were stimulated with PMA and Ionomycin as a positive control or co‐cultured with target cells. Degranulation was examined by CD107a expression (Figure S5D). Upon contact with autologous B cells, CD19^+^ Mec‐1 cells and CD19^+^ Nalm‐6 cells, CAR T cells showed increased CD107a expression compared to mock‐transduced T cells (Figure S5D). To further confirm CAR‐mediated cytotoxicity, CAR T cells and mock‐transduced T cells were co‐cultured with target cells and analyzed by flow cytometry. CAR T cells exhibited strong cytolytic activity against autologous B cells, CD19^+^ Mec‐1 cells, and CD19^+^ Nalm‐6 cells (Figure S5E). The cytolytic activity was pronounced against Mec‐1 and Nalm‐6 cells compared to autologous B cells (Figure S5E), indicating that CAR‐mediated cytotoxicity depends on CD19 expression levels, supporting previous findings.^27^ As expected, mock‐transduced T cells showed only background levels of cytolysis. Similarly, CAR T cells only exhibited baseline cytotoxicity when co‐cultured with the CD19^−^ U937 non‐B‐cell line (Figure S5E). Live cell imaging confirmed specific CAR T‐cell‐mediated lysis of primary CLL cells in co‐culture with stromal contact. HD‐derived CAR T cells reached maximal lysis after approximately 9 h, whereas mock‐transduced T cells induced only low levels of CLL cell apoptosis (Figure S5F).

To evaluate CD19‐dependent proliferation, CAR T cells and mock‐transduced T cells were co‐cultured with autologous primary B cells for 4 days. CAR T cells exhibited enhanced proliferation compared to the corresponding mock‐transduced counterparts when co‐cultured with autologous B cells. Consistently, the percentages of CAR^+^ T cells increased in the CD3^+^ T‐cell population after 4 days of target cell contact (Figure S5G).

### CLL‐ and ALL‐derived CAR T cells exhibit distinct CD4/CD8 ratios post‐transduction

**Figure 1:**
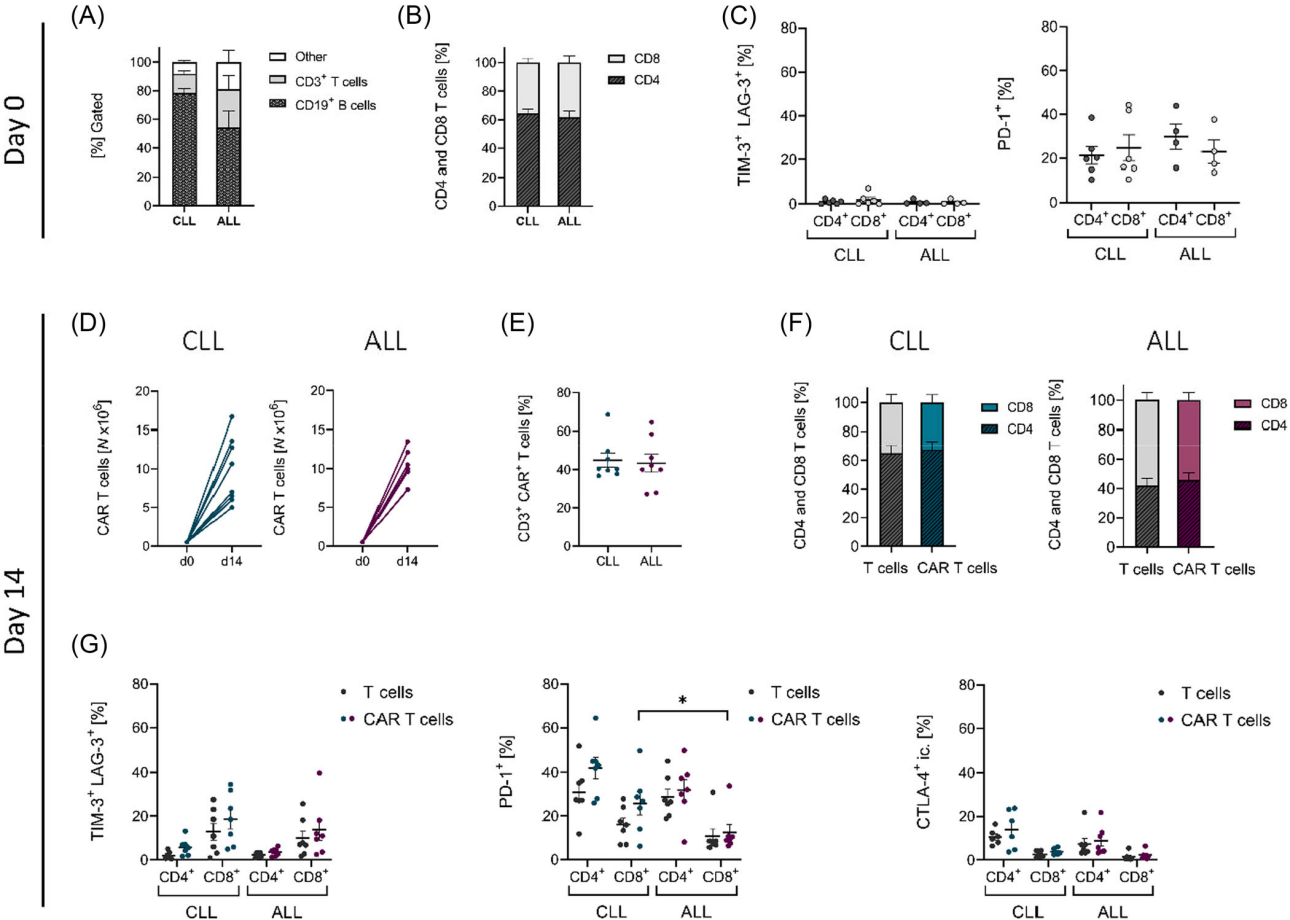
**Characterization of chronic lymphocytic leukemia (CLL) and acute lymphoblastic leukemia (ALL) patient‐derived T cells prior to and after transduction. (A)** Cellular composition of peripheral blood mononuclear cells (PBMCs) derived from CLL (*n* = 8) and ALL (*n* = 8) patients before T‐cell enrichment. The percentages of viable PBMCs are shown, subdivided into CD3^+^ T cells, CD19^+^ B cells, and other PBMCs. **(B)** Percentages of CD4^+^ and CD8^+^ T cells, enriched from CLL‐derived (*n* = 7) and ALL‐derived (*n* = 8) PBMCs. **(C)** Percentages of exhausted TIM‐3^+^ and LAG‐3^+^ (left panel) and PD‐1^+^ (right panel) CD4^+^ and CD8^+^ T cells, after T‐cell enrichment from CLL‐derived (*n* = 6) and ALL‐derived (*n* = 4) PBMCs. Data show individual values with indication of the mean. **(D)** In vitro expansion per well of transduced CD19 chimeric antigen receptor (CAR) T cells derived from CLL (*n* = 8) and ALL (*n* = 8) patients on Day 14 after transduction. **(E)** Transduction efficiency of CLL and ALL patient‐derived CAR T cells (*n* = 8) assessed on Day 14 after transduction. Data show individual values with indication of the mean. **(F)** Percentages of CD4^+^ and CD8^+^ T cells of CLL and ALL patients (*n* = 8) within the CD3^+^ mock‐transduced (gray scale) and CD3^+^ CAR T‐cell population (colored) on Day 14 after transduction. Data are presented as mean ± SEM. **(G)** Percentages of exhausted TIM‐3^+^ and LAG‐3^+^ (left panel), PD‐1^+^ (middle panel), and intracellular CTLA‐4^+^ (right panel) CD4^+^ and CD8^+^ T cells within the mock‐transduced T‐cell and CAR T‐cell population of CLL and ALL patients on Day 14 post‐transduction. Data show individual values with indication of the mean. Data were analyzed by Šidák's multiple comparisons test. Significance is indicated by *P = 0.05.

PBMCs were isolated from eight patients with CLL and eight patients with ALL and analyzed for their B and T‐cell composition prior to T‐cell enrichment. As expected, B cells were the predominant cell type in PBMCs from both CLL and ALL patients. However, on average, ALL patient‐derived PBMCs contained a lower proportion of B cells compared to those from CLL patients (Figure 1A). After T‐cell enrichment, the ratio of CD4^+^ to CD8^+^ T cells was comparable between CLL‐ and ALL‐derived T cells (Figure 1B). Additionally, the co‐expression of exhaustion markers TIM‐3 (*T‐cell immunoglobulin and mu*cin*‐domain containing‐3*) and LAG‐3 (*Lymphocyte activation gene‐3*), as well as PD‐1 (*Programmed cell death protein‐1*), was similar between ALL‐ and CLL‐derived T cells (Figure 1C). Analogous to HD‐derived T cells, CD4^+^ and CD8^+^ T cells isolated from PBMCs of CLL and ALL patients were transduced with a lentiviral vector encoding a CD19‐directed CAR. Control T cells were mock‐transduced under the same conditions. CAR T cells from both patient groups exhibited rapid expansion until harvest on Day 14 (Figure 1D). All samples showed similar levels of CD19 CAR expression (Figure 1E). While the CD4^+^/CD8^+^ T‐cell ratio remained stable after transduction in CLL‐derived T cells, the ratio in ALL‐derived T cells shifted, with an increase in both CD8^+^ T cells (38.4% ± 4.4 vs. 58.1% ± 4.9, P < 0.01 Mann–Whitney *U* test) and CD8^+^ CAR T cells (38.4% ± 4.4 vs. 54.1% ± 5.0, P < 0.05 Mann–Whitney *U* test, Figure 1B,F). The transduction process slightly increased the co‐expression of exhaustion markers TIM‐3 and LAG‐3 in CD8^+^ T cells (Figure 1G, left panel), likely due to T‐cell stimulation prior to transduction. However, the proportion of T cells expressing these markers remained low (<20%) across all subsets. PD‐1 expression remained largely unchanged after transduction, with the exception of a modest increase in PD‐1^+^ CD4^+^ CLL‐derived T cells and CAR T cells, and a decrease in PD‐1^+^ CD8^+^ ALL‐derived T cells and CAR T cells (Figure 1G, middle panel). Notably, CLL‐derived CD8^+^ CAR T cells exhibited significantly higher PD‐1 expression than their ALL‐derived counterparts after transduction and expansion (CLL: 25.8% ± 5.2 vs. ALL: 12.5% ± 3.6, P < 0.05, Figure 1G, middle panel). Finally, intracellular CTLA‐4 (*Cytotoxic T‐lymphocyte‐associated Protein 4*) expression was slightly increased in CLL‐derived CAR T cells compared to ALL‐derived CAR T cells, although expression levels remained low overall (Figure 1G, right panel).

### Efficient infiltration but reduced target engagement of CLL‐derived CAR T cells in 3D conditions

We developed a scaffold‐based 3D system consisting of primary malignant B cells from CLL or ALL patients, cultured on human HS‐5 BMSCs along with autologous CAR T cells. This system enables immune cells and BMSCs to interact in multiple dimensions, facilitating dynamic, relevant cell–cell communication. Within this 3D model, we can distinguish between peripheral and core regions. As previously shown, CLL cells gain a survival advantage by actively manipulating surrounding BMSCs through direct cellular interactions, especially in the core regions of the 3D scaffold. Moreover, 3D conditions can lead to altered therapeutic effects compared to 2D cultivation, which was previously shown for several clinically used inhibitors.^28^ By providing a more physiologically relevant environment, the 3D model allows the study of CAR T‐cell functionality in an autologous setting, enabling the identification of differences between ALL‐ and CLL‐derived CAR T cells within these protected stromal niches.

**Figure 2:**
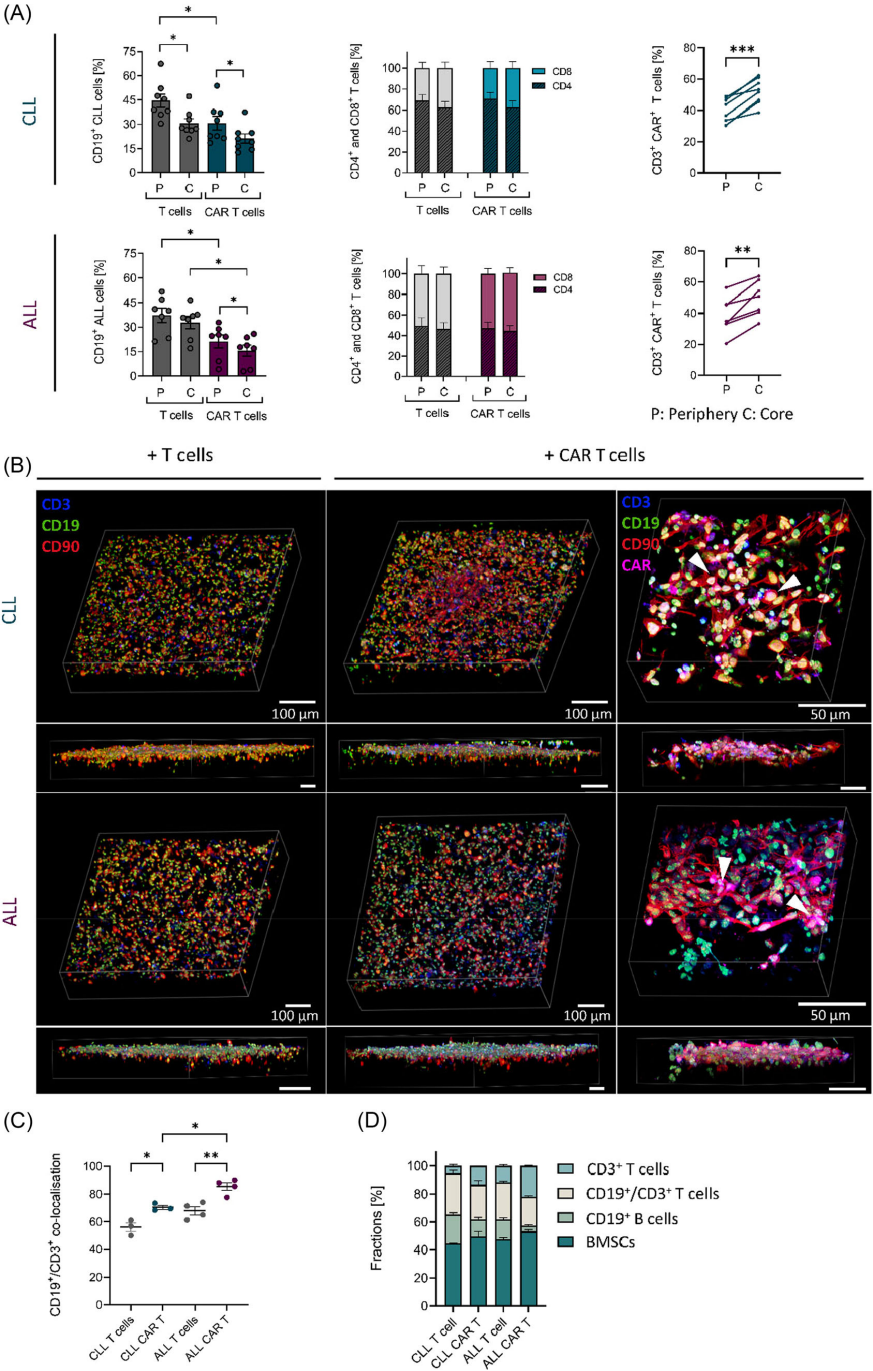
**Cellular distribution of immune cells and bone marrow‐derived stromal cells (BMSCs) in the autologous chronic lymphocytic leukemia (CLL) and acute lymphoblastic leukemia (ALL) three‐dimensional (3D) co‐culture system. (A)** Percentages of CD19^+^ malignant B cells, viable CD4^+^ and CD8^+^ T cells, and viable CD3^+^ CAR^+^ T cells in the peripheral (P) and the core (C) regions of the 3D co‐cultures with primary CLL cells (upper panels, *n* = 8) and ALL cells (lower panels, *n* = 7) after co‐culture with mock‐transduced autologous T cells or chimeric antigen receptor (CAR) T cells for 18 h. Data were analyzed by Tukey's multiple comparisons test and paired *t*‐test for comparisons between periphery and core. A gating strategy is shown in Figure S2. **(B)** Representative 3D reconstructions of co‐cultures of CLL (upper panel) and ALL (lower panel) cells with autologous T cells (left panel) or CAR T cells (right panel) for 18 h. The visualizations show the distribution of different cell types within the scaffold‐based 3D co‐culture model. *z*‐Stacks of the stained 3D co‐cultures including BMSCs (CD90, red), B cells (CD19, green), and T cells (CD3, blue) were analyzed using Fiji software (upper panel: number of stacks from left to right: 114, 114, and 207; size of stacks: 1.04, 1.04, and 0.4 µm; lower panel: number of stacks from left to right: 119, 158, and 162; size of stacks: 1.04, 1.04, and 0.34 µm). The higher magnification on the right highlights the co‐localization of CAR T cells (CAR^+^, magenta) with malignant B cells (magenta and green overlap, white arrows), which strongly co‐localize with mesenchymal stromal cells (green and red overlap). Corresponding 3D reconstructions showing only CD3⁺/CAR⁺ T cells and CD19⁺ B cells are provided in Figure S6A. **(C)** Quantitative image analysis of CLL (*n* = 3) and ALL (*n* = 4) patient‐derived 3D scaffolds demonstrates the fraction of CD19^+^ B cells co‐localized with CD3^+^ T cells (CD19^+^/CD3^+^ cells). Data points represent means of analyzed images per patient, with indication of the mean, and statistical comparisons performed by Tukey's multiple comparisons test. The number of analyzed *z*‐stacks and regions for each patient is provided in Figure S6B. **(D)** Quantitative image analysis of cellular composition within CLL and ALL patient‐derived 3D scaffolds across the individual patient measurements. Data are presented as mean ± SEM. Significance is indicated by *P = 0.05, **P = 0.01, and ***P = 0.001.

Flow cytometric analysis of autologous 3D co‐cultures with either mock‐transduced T cells or CAR T cells demonstrated the distribution of CD19^+^ malignant B cells and CD3^+^ T cells between the periphery and core regions of the 3D model (Figure 2A). In CLL‐derived co‐cultures with mock‐transduced T cells, the percentage of CD19^+^ malignant B cells was higher in the periphery compared to core regions (Figure 2A, upper left panel), while ALL‐derived co‐cultures showed a more balanced distribution of malignant B cells between both regions (Figure 2A, lower left panel). As expected, treatment with autologous CAR T cells led to a reduction of CD19^+^ malignant B cells in both models, which was more pronounced in the core of ALL‐derived co‐cultures than in the CLL setting (mean difference: −17.2% ± 5.5 ALL; 9.3% ± 5.0 CLL; Figure 2A, left panels). The CD4/CD8 T‐cell ratio after co‐culture remained consistent with the proportions observed at Day 14 post‐expansion. In line with earlier results, ALL‐derived T cells exhibited a high proportion of CD8^+^ T cells, whereas CD4^+^ T cells were predominant in CLL‐derived T‐cell populations (Figures 1F and 2A, middle panel). Analysis of CAR T cells within the CD3^+^ population revealed a higher proportion of CAR T cells localized in the core regions in both models, indicating efficient migratory capacity into protected stromal areas (Figure 2A, right panel).

To visualize the spatial arrangement of malignant B cells, T cells, and BMSCs within the 3D co‐culture, *z*‐stack data from confocal laser microscopy were reconstructed into 3D representations (Figure 2B). Consistent with previous observations, the 3D models revealed pronounced co‐localization of malignant B cells with BMSCs, with stromal cells forming a structural network by re‐establishing the scaffold architecture through strong adherence.^26^ In accordance with flow cytometric analysis, immune cells demonstrate migration ability within the scaffold. In co‐cultures with autologous CAR T cells derived from either CLL or ALL patients, T cells were frequently in close contact with malignant B cells situated on stromal cells, whereas mock‐transduced T cells appeared more randomly distributed (Figure 2B). Higher magnification further highlights the co‐localization of patient‐derived CAR T cells with target B cells (Figure 2B, white arrows). Quantitative image analysis confirmed enhanced co‐localization between malignant B cells and T cells in 3D co‐culture with autologous CAR T cells compared to mock‐transduced T cells (Figure 2C). Notably, co‐localization was more pronounced in the ALL‐derived CAR T‐cell co‐culture, indicating more efficient CAR T‐cell‐target cell interaction, a prerequisite for effective cytotoxicity. To complement spatial localization data, we quantified the overall cellular composition within the 3D scaffolds using image‐based analysis (Figure 2D). The distribution of stromal cells (BMSCs), CD19⁺ malignant B cells, CD3⁺ T cells, and CD19⁺/CD3^+^ co‐localized cells was assessed across co‐cultures. In both CLL and ALL samples, BMSCs comprised a consistent scaffold‐resident population. In line with flow cytometry and microscopy findings, CD19⁺ B cells were reduced upon CAR T‐cell treatment, with a more pronounced effect observed in ALL‐derived co‐cultures. Additionally, the proportion of CD3⁺ T cells was comparable across conditions, while CD19⁺/CD3^+^ co‐localized cells were enriched in CAR T‐cell co‐cultures, indicating increased target engagement. These data confirm the effective infiltration and interaction of CAR T cells with malignant B cells in the 3D TME model.

### CLL‐derived CAR T cells show strong activation but reduced proliferation in 3D co‐culture

**Figure 3:**
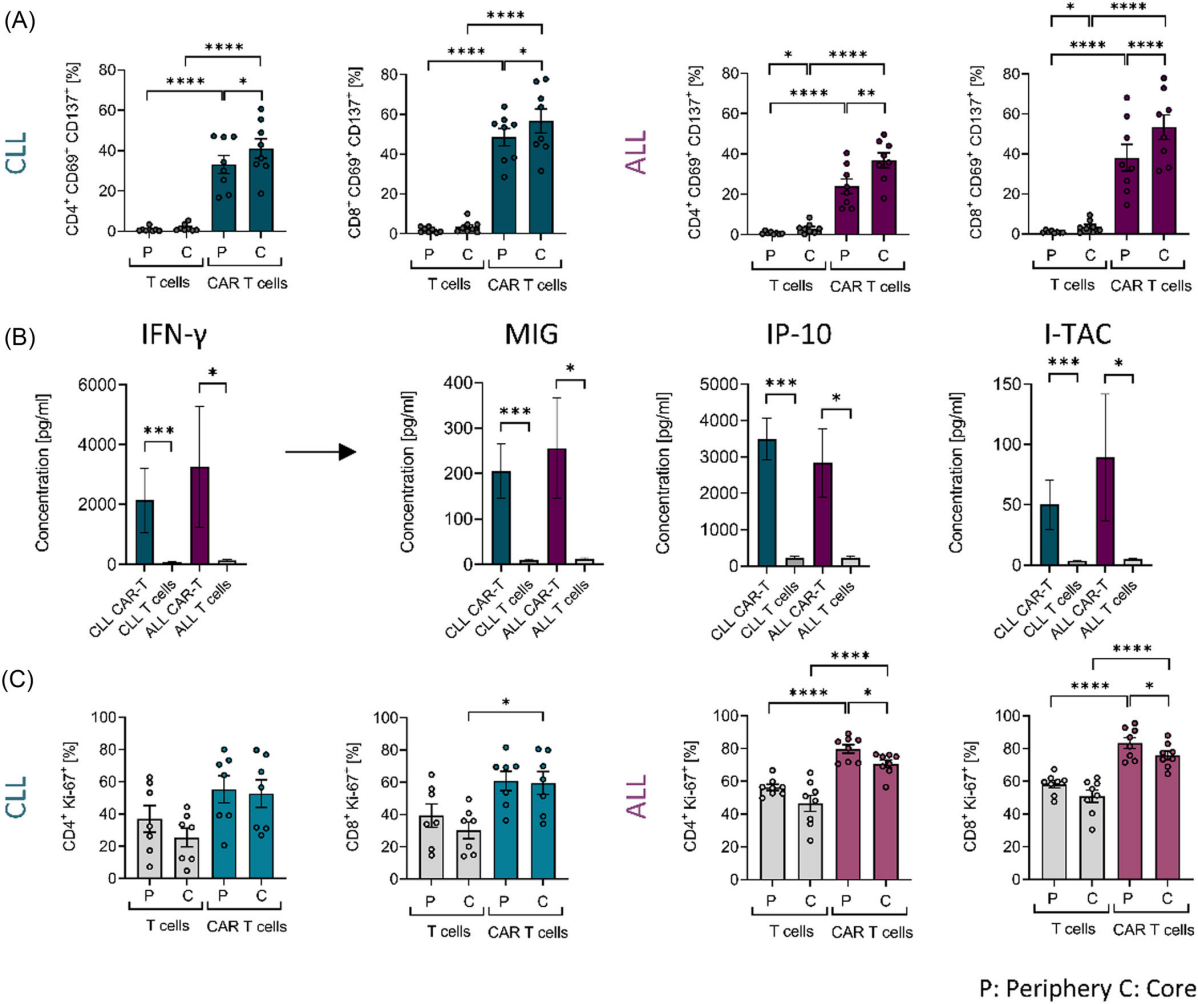
**Activation of chronic lymphocytic leukemia (CLL) and acute lymphoblastic leukemia (ALL) patient‐derived chimeric antigen receptor (CAR) T cells and proliferative response in three‐dimensional (3D) co‐culture. (A)** Percentages of early activated CD69^+^ and CD137^+^ CLL (*n* = 8) or ALL (*n* = 8) patient‐derived mock‐transduced T cells and CAR T cells after 18 h of 3D co‐culture. **(B)** Release of interferon‐γ (IFN‐γ) and IFN‐γ‐induced proteins MIG (monokine induced by interferon gamma), IP‐10 (interferon gamma‐induced protein 10), and I‐TAC (interferon‐inducible T‐cell alpha chemoattractant) after 24 h of 3D co‐culture with CLL‐ or ALL‐derived mock‐transduced T cells or CAR T cells, measured by multiplex immunoassay of cell culture supernatants. **(C)** Proliferative response of CLL and ALL patient‐derived mock‐transduced T cells and CAR T cells after 4 days of 3D co‐culture, assessed by Ki‐67 expression. Data were analyzed by Tukey's multiple comparisons test and paired *t*‐test for comparisons between peripheral and core regions (A, C) or Mann–Whitney *U* test (B). Bars represent mean ± SEM. Significance is indicated by *P = 0.05, **P = 0.01, ***P = 0.001, and ****P = 0.0001. C, core; P, periphery.

Antigen‐specific activation upon target recognition represents an essential functional parameter of CAR T cells. To investigate potential differences in activation between ALL‐ and CLL‐derived CAR T cells, we analyzed the target response in autologous 3D co‐cultures with malignant B cells using flow cytometric analysis. CAR T cells from both CLL and ALL patients exhibited strong activation, as indicated by the co‐expression of the early activation marker CD69 and the antigen‐specific activation marker CD137 (Figure 3A). Notably, activation levels of CAR T cells were particularly high within the core regions of the 3D co‐cultures. For both CLL‐ and ALL‐derived CAR T cells, the proportion of activated cells was higher within the CD8^+^ T‐cell subset (Figure 3A). To further confirm activation, we measured the release of interferon‐gamma (IFN‐γ) in co‐culture supernatants using multiplex immunoassays with either autologous mock‐transduced T cells or CAR T cells. A strong IFN‐γ response was detected in both CLL and ALL co‐cultures, accompanied by elevated levels of downstream effector—chemokines, including *monokine induced by interferon gamma* (MIG), *interferon gamma‐induced protein 10* (IP‐10), and *interferon‐inducible T‐cell alpha chemoattractant* (I‐TAC) (Figure 3B). In contrast, mock‐transduced patient‐derived T cells showed minimal co‐expression of activation markers and no detectable IFN‐γ response in 3D co‐cultures, underscoring the antigen‐specific activation of CD19 CAR T cells upon target cell engagement within the 3D co‐culture systems (Figure 3A,B).

To determine whether patient‐derived CAR T cells remain activated over time within the 3D co‐culture, the co‐expression of late activation markers HLA‐DR and CD38 was assessed after 4 days of co‐culture. Both CLL‐ and ALL‐derived CAR T cells showed increased co‐expression of these markers, particularly within the CD8^+^ T‐cell subset, implicating that a high activation status is sustained throughout the co‐culture period (Figure S7). However, despite this sustained activation, the proportion of proliferating Ki‐67⁺ CLL‐derived CAR T cells was significantly lower compared to their ALL‐derived counterparts in the periphery of the 3D co‐culture (CD4^+^ CAR T cells: CLL 55.3% ± 8.4 vs. ALL 79.7% ± 2.6; CD8^+^ CAR T cells: CLL 60.9% ± 6.0 vs. ALL 83.4% ± 3.3; P < 0.05 Mann–Whitney *U* test, Figure 3C), indicating a reduced proliferative capacity of CLL‐derived CAR T cells. This emphasizes that antigen‐induced activation alone does not fully determine CAR T‐cell functionality, but rather the result of a complex interplay between pro‐inflammatory signals and immunosuppressive, anti‐inflammatory components within the TME.

### Reduced cytotoxicity and decreased secretion of cytotoxic proteins by CLL‐derived CAR T cells

**Figure 4:**
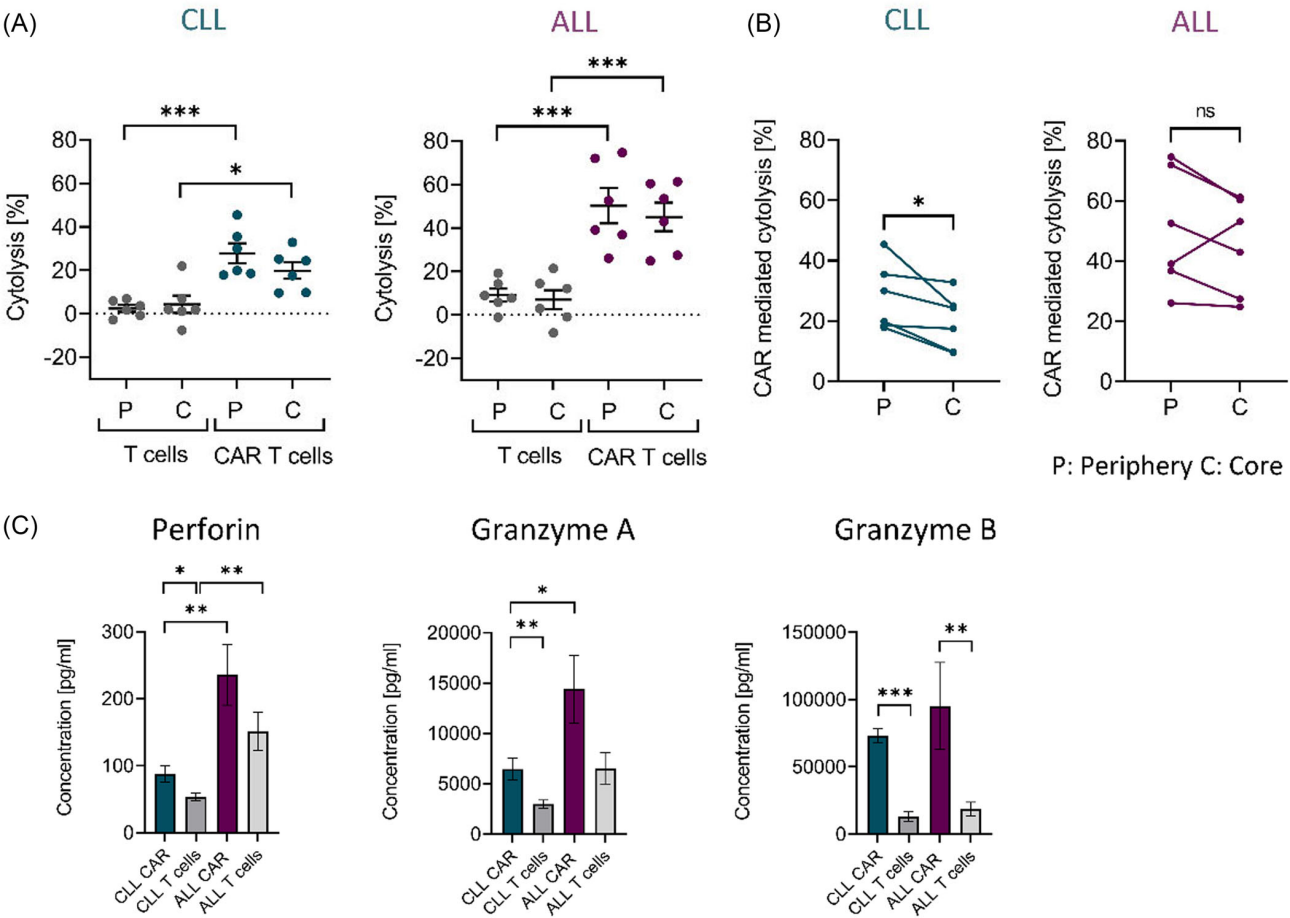
**Cytolytic activity of chronic lymphocytic leukemia (CLL) and acute lymphoblastic leukemia (ALL) patient‐derived chimeric antigen receptor (CAR) T cells in three‐dimensional (3D) co‐culture. (A)** 3D co‐cultures with bone marrow‐derived stromal cells (BMSCs) and primary CLL or ALL cells were co‐cultured with autologous CAR T cells or mock‐transduced T cells for 18 h (*n* = 6). Cytolysis by effector cells was defined as (% live cells [target cells alone] − % live cells [sample of interest])/% live cells (target cells alone) × 100. Data show individual values with indication of the mean, and were analyzed by Tukey's multiple comparisons test. **(B)** Comparison of cytolysis mediated by CAR T cells between the periphery and core region of CLL and ALL patient‐derived 3D co‐cultures after 18 h (*n* = 6). Data were analyzed by a paired *t*‐test. **(C)** Release of cytotoxic proteins in CLL and ALL 3D co‐culture after 24 h of co‐culture with autologous CAR T cells or mock‐transduced T cells, measured by multiplex immunoassay of cell culture supernatants (*n* = 8). Data were analyzed by the Mann–Whitney *U* test. Data are presented as mean ± SEM. Significance is indicated by *P = 0.05, **P = 0.01, and ***P = 0.001. C, core; P, periphery.

In order to determine cytotoxicity of patient‐derived autologous CAR T cells in 3D conditions, mock‐transduced T cells or CAR T cells were co‐cultured for 18 h at an E:T ratio of 1:1. Despite showing comparable activation levels, CAR T cells from CLL and ALL patients exhibited significant differences in cytolytic activity. CAR T cells derived from CLL patients displayed reduced cytolysis of malignant B cells compared to CAR T cells derived from ALL patients, in both peripheral and core regions of the scaffold (P: 27.9% ± 4.6 CLL vs. 50.2% ± 8.1 ALL, P < 0.05; C: 19.9% ± 3.8 CLL vs. 45.1% ± 6.6 ALL, P < 0.05 Mann–Whitney *U* test, Figure 4A). This disparity was particularly evident in the core regions, where CLL‐derived CAR T cells exhibited lower cytotoxicity than in the periphery (mean difference: −8.0% ± 2.8, P < 0.05; Figure 4B). Additionally, multiplex immunoassays revealed significantly lower levels of Perforin and Granzyme A in CLL‐derived CAR T‐cell co‐cultures compared to those derived from ALL patients (Figure 4C). These findings suggest that the diminished cytotoxic potential of CLL‐derived CAR T cells may be attributed to impaired effector molecule release in the 3D microenvironment.

### Increased exhaustion phenotype of CAR T cells derived from CLL patients

**Figure 5:**
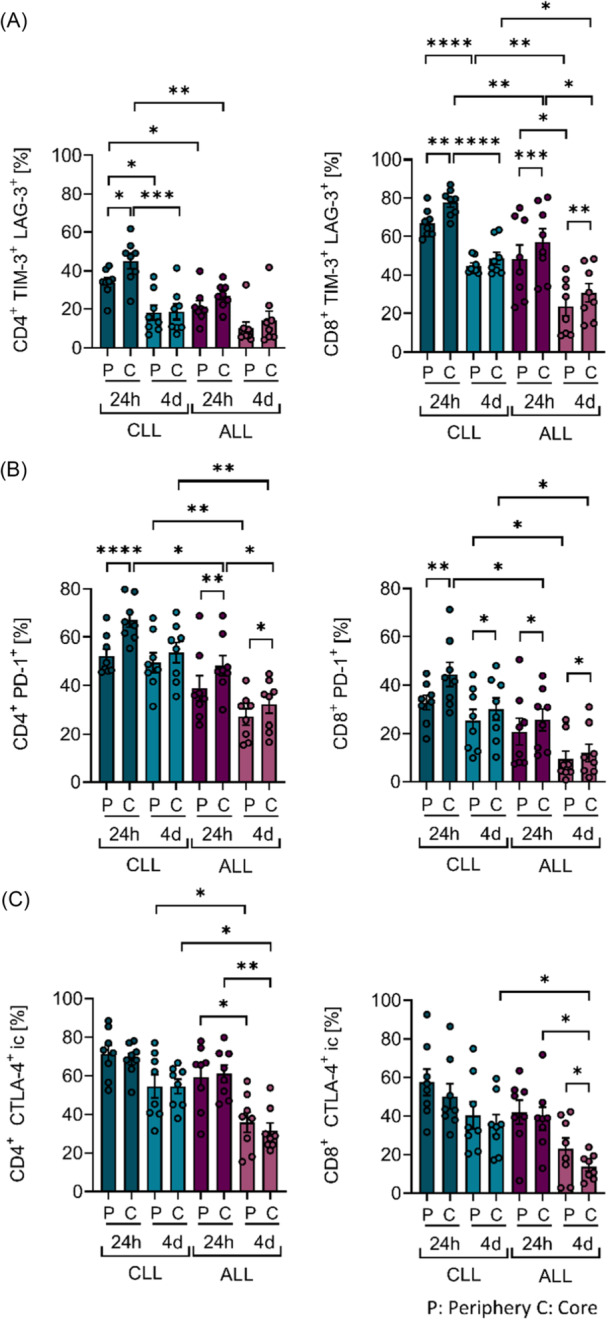
**Exhaustion of chronic lymphocytic leukemia (CLL) and acute lymphoblastic leukemia (ALL) patient‐derived chimeric antigen receptor (CAR) T cells in three‐dimensional (3D) co‐culture.** Percentages of exhausted **(A)** TIM‐3^+^ and LAG‐3^+^, **(B)** PD‐1^+^, and **(C)** intracellular CTLA‐4^+^ CD4^+^ and CD8^+^ CLL and ALL patient‐derived CAR T cells after 24 h and 4 days of autologous 3D co‐culture (*n* = 8). Data were analyzed by Tukey's multiple comparisons test, with group comparisons performed separately for each time point and within‐group comparisons between time points. Paired *t*‐test was used for comparisons between peripheral and core regions. Bars represent mean ± SEM. Significance is indicated by *P = 0.05, **P = 0.01, ***P = 0.001, and ****P = 0.0001. C, core; P, periphery.

To gain a deeper understanding of the functional limitations of CAR T cells in CLL, the exhaustion phenotype of mock‐transduced T cells and CAR T cells derived from CLL or ALL patients was assessed in 3D co‐culture. Multiple exhaustion markers were analyzed by flow cytometry. In our 3D model, the co‐inhibitory receptors TIM‐3 and LAG‐3, which are transiently upregulated upon T‐cell activation, were elevated after 24 h compared to mock‐transduced T cells (Figure S8A). Notably, CLL‐derived CAR T cells displayed a higher proportion of TIM‐3⁺ and LAG‐3⁺ cells than those derived from ALL patients. This effect was most pronounced within the CD8⁺ T‐cell subset, particularly in core regions of the scaffold (Figure 5A). After 4 days of co‐culture, the co‐expression of these markers declined, reflecting the dynamic regulation of exhaustion marker expression in response to activation (Figure 5A). However, CD8⁺ CAR T cells from CLL patients continued to express significantly higher levels of TIM‐3⁺LAG‐3⁺ than their ALL counterparts after 4 days of co‐culture (mean difference in P: −21.4% ± 5.5; in C: −18.1% ± 5.5, P < 0.05). Furthermore, after 24 h of co‐culture, CAR T cells derived from CLL and ALL patients displayed increased levels of PD‐1⁺ and intracellular CTLA‐4⁺ cells compared to mock‐transduced T cells, particularly within the CD4⁺ subset (Figure S8B,C). Expression levels of both exhaustion markers were notably higher in CLL‐derived CAR T cells compared to ALL‐derived counterparts within the core regions after 4 days of co‐culture (Figure 5B,C). In addition, the proportion of PD‐1 expressing CAR T cells was consistently higher in the core regions than in the periphery after 24 h, consistent with the increased activation previously observed in the core regions (Figures 5B and 3A). Interestingly, PD‐1 and CTLA‐4 expression declined in ALL‐derived CD4^+^ CAR T cells in the core regions after 4 days of co‐culture, indicating a partial recovery from exhaustion. In contrast, no significant decrease was observed in CLL‐derived CD4^+^ and CD8^+^ CAR T cells, implying a persistently increased exhaustion phenotype in CLL CAR T cells (Figure 5B,C). Notably, the proportion of PD‐1⁺ cells was also elevated within the CD8⁺ CAR T‐cell subset in CLL co‐culture. The increased frequency of CD8^+^ PD‐1^+^ CLL‐derived CAR T cells was already evident after expansion (Figure 1G, middle panel). Moreover, a higher percentage of terminal exhausted TIM‐3^+^ LAG‐3^+^ PD‐1^+^ CAR T cells derived from CLL patients was observed compared to those derived from ALL patients (Figure S8D). Across all analyzed markers, CLL‐derived CAR T cells exhibited consistently higher exhaustion levels after 4 days of co‐culture, underscoring a more pronounced and sustained exhaustion phenotype under 3D culture conditions (Figure 5A–C).

### CLL patient‐derived CAR T cells display a further differentiated memory phenotype than ALL patient‐derived CAR T cells

Since the antitumor efficacy and long‐term persistence of CAR T cells are closely linked to their differentiation status,^9^ we next assessed the memory phenotype of CLL‐ and ALL patient‐derived CAR T cells before and after transduction, as well as following 3D co‐culture.

**Figure 6:**
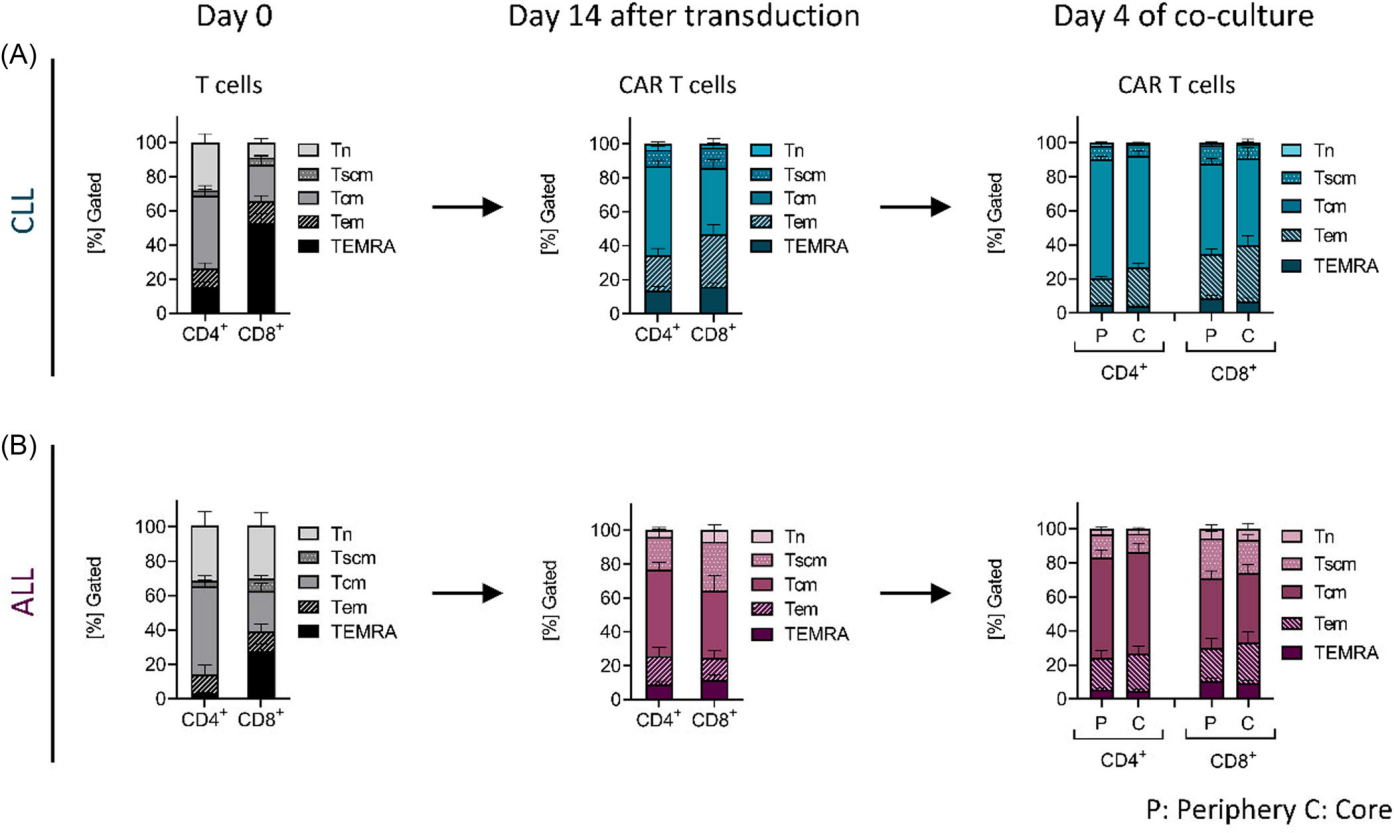
**Differentiation status of chronic lymphocytic leukemia (CLL) and acute lymphoblastic leukemia (ALL) patient‐derived T cells and chimeric antigen receptor (CAR) T cells before and after transduction and following three‐dimensional (3D) co‐culture.** Fractions of memory phenotypes of enriched CD4^+^ and CD8^+^ T cells (gray‐scaled) from **(A)** CLL (*n* = 8) and **(B)** ALL (*n* = 8) patient‐derived peripheral blood mononuclear cells (PBMCs) on Day 0 before transduction and of CAR T cells (colored) on Day 14 after transduction and Day 4 of co‐culture. Naïve T cells (Tn, CCR7^+^, CD95^−^, and CD45RO^−^), stem cell‐like memory T cells (Tscm, CCR7^+^, CD95^+^, and CD45RO^−^), central memory T cells (Tcm, CCR7^+^, CD45RO^+^), effector memory T cells (Tem, CCR7^−^, CD45RO^+^), and Tem re‐expressing CD45RA (TEMRA, CCR7^−^, CD45RO^−^) were determined by flow cytometry. A representative gating strategy is shown in Figure S9C. Bars represent mean ± SEM. C, core; P, periphery.

Following T‐cell enrichment from patient‐derived PBMCs, the CD4^+^ compartment in both CLL‐ and ALL‐derived T cells was predominantly composed of naïve T cells (Tn) and central memory T cell (Tcm) subsets, resulting in a similar composition between the two patient groups (Figure 6A,B, left panels). In contrast, CD8^+^ T cells from CLL patients were characterized by a high level of TEMRAs (terminally differentiated effector memory cells re‐expressing CD45RA) and contained a markedly reduced fraction of Tn cells compared to both their CD4^+^ counterparts and CD8^+^ T cells from ALL patients (Figure 6A, left panel). This skewing toward TEMRA cells in CLL‐derived CD8^+^ T cells suggests a higher level of senescence within this T‐cell subset.

After transduction, the proportion of TEMRA cells declined, likely reflecting the preferential expansion of subsets with higher proliferative potential or depletion of terminally differentiated cells during T‐cell stimulation. In addition, stimulation resulted in a nearly complete loss of Tn cells in CLL‐derived mock‐transduced T cells and CAR T cells (Figures 6A and S9A, middle panel). By contrast, the stem cell‐like memory T cell (Tscm) subset increased more prominently in ALL‐derived T cells (Figure 6B, middle panel), likely supported by the IL‐7 and IL‐15 supplementation during expansion, which also favors a Tcm phenotype.^29^, ^30^, ^31^ After transduction, CLL‐derived mock‐transduced T cells and CAR T cells exhibited a more differentiated phenotype compared to those from ALL patients, particularly in the CD8^+^ compartment (Figures 6A,B and S9A,B, middle panels). In CLL‐derived CAR T cells, Tem and T cm were the predominant subsets, while ALL‐derived cells retained less differentiated phenotypes such as Tn and Tscm (Figure 6A,B, middle panels).

Following 3D co‐culture, the differentiation status of CAR T cells shifted toward further differentiated phenotypes. In both CLL‐ and ALL‐derived CAR T cells, the CD8⁺ compartment showed an enrichment of Tem, whereas the CD4⁺ compartment predominantly exhibited a Tcm increase (Figure 6A,B, right panels). Consistent with post‐transduction observations CLL‐derived CAR T cells retained a more differentiated profile, with high frequencies of Tem and Tcm, while ALL‐derived CAR T cells preserved higher levels of Tn (CD4^+^ CAR T cells in C: CLL 0.9% ± 0.1 vs. ALL 2.9% ± 0.7; CD8^+^ CAR T cells in C: CLL 0.8% ± 0.3 vs. ALL 6.3% ± 2.8; P < 0.05 Mann–Whitney *U* test) and Tscm (CD8^+^ CAR T cells in P: CLL 10.7% ± 2.3% vs. ALL 23.2% ± 4.0; in C CLL: 8.6% ± 2.8 vs. ALL 19.5% ± 3.1; P < 0.05 Mann–Whitney *U* test). A similar pattern was observed in CD8^+^ mock‐transduced counterparts. As expected, Tn and Tscm were more abundant in mock‐transduced T cells than in CAR T cells, both in CLL and ALL settings (Figure S9A,B).

### Combination treatments with various inhibitors enhance cytotoxicity and reduce CAR T‐cell exhaustion in CLL 3D co‐cultures

To evaluate whether pharmacologic modulation could enhance cytotoxicity in our autologous CLL co‐culture, we tested clinically approved CLL therapeutics for their capacity to improve cytotoxicity in combination with CAR T cells. CLL co‐cultures were treated with the BTK inhibitor Acalabrutinib, the PI3Kδ inhibitor Idelalisib, or the Bcl‐2 inhibitor ABT199 for 72 h, with autologous mock‐transduced T cells or CAR T cells added after 48 h and incubated for an additional 24 h in the presence of respective inhibitors. All three inhibitors reduced the viability of primary CLL cells in co‐cultures without T cells, with mock‐transduced T cells, and in combination with CAR T cells, in both peripheral and core regions of the 3D model (Figure S10A). As expected, CAR T‐cell treatment alone decreased CLL viability, but cytotoxic effects were more pronounced in the periphery than in the core. In contrast, co‐treatment with targeted agents further diminished viability even in the core, highlighting their potential to overcome stromal‐mediated resistance (Figure S10A). Among the tested compounds, co‐treatment with ABT199 showed the strongest effect (P: −25.9% ± 2.0; C: −21.6% ± 1.6), followed by Idelalisib (P: −21.5% ± 0.9; C: −16.2% ± 1.1) and Acalabrutinib (P: −18.7% ± 0.5; C: −14.2% ± 1.0). Notably, none of the agents negatively impacted CAR T‐cell viability (Figure S10C).

**Figure 7:**
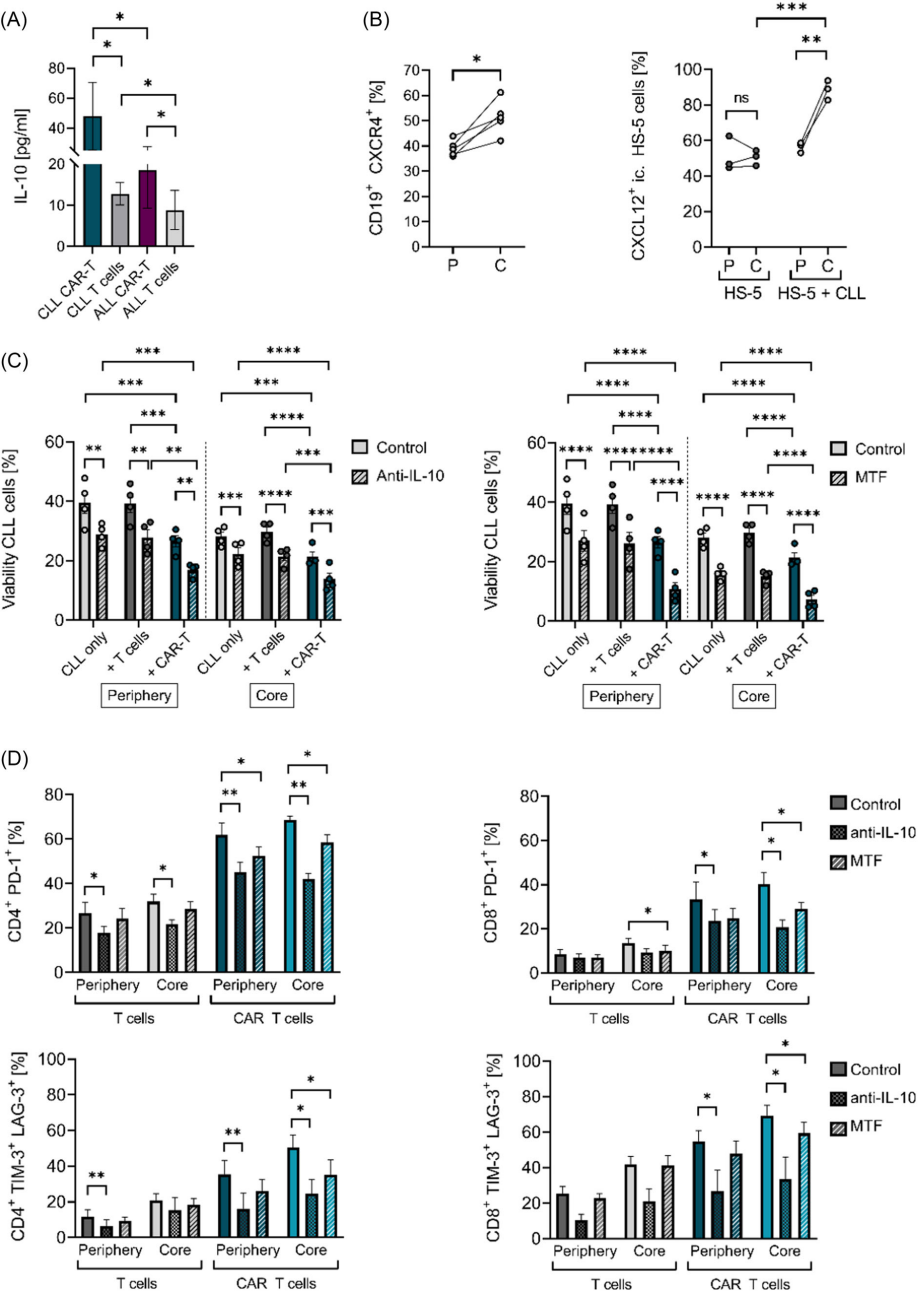
**Blockade of interleukin‐10 (IL‐10) and CXCR4 in chronic lymphocytic leukemia (CLL)‐derived three‐dimensional (3D) co‐cultures to enhance cytotoxicity of chimeric antigen receptor (CAR) T‐cell treatment. (A)** Release of IL‐10 in CLL and acute lymphoblastic leukemia (ALL) 3D co‐culture after 24 h of co‐cultivation with autologous mock‐transduced T cells or CAR T cells, measured by IL‐10 enzyme‐linked immunosorbent assay (ELISA) (*n* = 8). Data were analyzed by the Mann–Whitney *U* test. **(B)** Percentages of CXCR4^+^ CLL cells in the periphery and core after 3 days of co‐culture (*n* = 5, left panel). Data were analyzed by paired *t*‐test (left panel). Percentages of intracellular CXCL12^+^ HS‐5 cells with and without CLL contact in 3D culture after 3 days (*n* = 3, right panel). Data were analyzed by Šidák's multiple comparisons test (two‐way analysis of variance [ANOVA]). **(C)** Viability of CLL cells after treatment of CLL co‐cultures (*n* = 4) with anti‐IL‐10 antibody (500 ng/mL) or Motixafortide (MTF, 15 µM) for 96 h. After 72 h of treatment, mock‐transduced T cells or CAR T cells were added. CLL co‐cultures without effector cells were used as a control; 0.15% dimethyl sulfoxide (DMSO) was used as a solvent control. Data were analyzed by Tukey's multiple comparison (two‐way ANOVA). **(D)** Percentages of exhausted CD4^+^ and CD8^+^ PD‐1^+^ (upper panel) and TIM‐3^+^ LAG‐3^+^ (lower panel) mock‐transduced T cells and CAR T cells after treatment of autologous CLL co‐cultures with anti‐IL‐10 or MTF as described in (C). Data were analyzed by a paired *t*‐test. Bars represent mean ± SEM. Significance is indicated by ns = not significant, *P = 0.05, **P = 0.01, ***P = 0.001, and ****P = 0.0001. C, core; P, periphery.

Given the functional impairments and pronounced exhaustion of CLL‐derived CAR T cells, we further investigated immunosuppressive mechanisms potentially responsible for these effects. As IL‐10 represents a central immunosuppressive mediator in the TME of various cancers, we examined its role in our 3D model.^32^, ^33^ Analysis of co‐culture supernatants by enzyme‐linked immunosorbent assay (ELISA) revealed elevated IL‐10 secretion in CLL‐derived co‐cultures with autologous mock‐transduced T cells compared to those derived from ALL (Figure 7A). This effect was even more pronounced in CLL‐derived co‐cultures with CAR T cells, which exhibited significantly higher IL‐10 levels than their ALL‐derived counterparts (Figure 7A). Since IL‐10 exhibits anti‐inflammatory effects, this may represent a mechanism that dampens CAR T‐cell activity, especially in the CLL co‐culture setting. Besides IL‐10, we targeted the CXCR4/CXCL12 axis, due to its important role in stromal‐dependent resistance to therapy, further contributing to an immunosuppressive TME, which may impair CAR T‐cell function in CLL. Flow cytometric analysis of 3D CLL‐derived co‐cultures confirmed upregulation of CXCR4 at the protein level, with a higher proportion of CXCR4⁺ CLL cells localized in the core region compared to the periphery (Figure 7B, left panel). Additionally, a greater percentage of intracellular CXCL12⁺ HS‐5 stromal cells was detected in the core after contact with CLL cells, indicating spatial regulation of stromal cells within the 3D co‐culture (Figure 7B, right panel).

In addition, we examined whether IL‐10 neutralization in our 3D CLL co‐cultures could reduce CLL cell survival and improve the efficacy of CAR T cells. IL‐10 neutralization led to reduced CLL cell viability across all culture conditions: in the absence of T cells, with mock‐transduced T cells, and in combination with CAR T cells (Figure 7C, left panel). Notably, the combination of IL‐10 blockade and CAR T‐cell treatment further enhanced cytotoxicity, with a more pronounced effect in the periphery (Figure 7C, left panel). ELISA confirmed full neutralization of IL‐10 in the co‐culture supernatant (Figure S4C).

To target the CXCR4/CXCL12 axis, we used the CXCR4 antagonist Motixafortide (MTF, BL‐8040), which is Food and Drug Administration (FDA)‐approved as a hematopoietic stem cell mobilizer in multiple myeloma, and tested whether combining MTF with CAR T cells potentiates cytotoxicity in our 3D model. MTF mono‐treatment already led to a reduction in CLL cell viability across all co‐culture conditions (Figure 7C, right panel). Interestingly, its effect was particularly strong in the core regions of the 3D model, suggesting better accessibility to stromal‐dense areas than other tested agents. Combined treatment with MTF and CAR T cells drastically enhanced cytotoxicity in both peripheral and core regions, indicating re‐sensitization of stromal‐protected CLL cells to CAR T‐cell treatment by mobilization of CLL cells from protected regions (Figure 7C, right panel). Blockade of CXCR4 on CLL cells was confirmed by flow cytometry (Figure S4D). Notably, CAR T‐cell viability remained unaffected by either MTF treatment or IL‐10 blockade (Figure S4A).

To determine the effects of co‐treatments on CAR T‐cell exhaustion, we analyzed the expression of TIM‐3, LAG‐3, and PD‐1 by flow cytometry. BCR signaling inhibitors and ABT199 reduced the percentage of exhausted CD4⁺ and CD8⁺ CAR T cells co‐expressing TIM‐3 and LAG‐3 or PD‐1, in both peripheral and core regions of the 3D model (Figure S10B). This effect was most pronounced with Idelalisib, which also lowered exhaustion levels in mock‐transduced control T cells (Figure S10B). MTF reduced exhaustion preferentially in core regions, likely due to decreased local immunosuppression following CXCR4 blockade. IL‐10 neutralization showed the most pronounced effect, significantly reducing exhaustion marker expression in core‐localized CAR T cells (Figure 7D). These findings underscore the potential of clinically approved inhibitors, along with IL‐10 and CXCR4 blockade, to support CAR T‐cell‐mediated cytotoxicity, alleviate CAR T‐cell exhaustion, and counteract the immunosuppressive and protective characteristics of the CLL microenvironment.

## DISCUSSION

Our study provides functional evidence that CAR T‐cell efficacy is shaped not only by disease‐intrinsic T‐cell properties but also by spatially organized signals within the TME. To investigate disease‐specific differences, we employed a scaffold‐based, autologous 3D co‐culture system that integrates primary malignant B cells, CAR T cells, and human BMSCs. This model enables spatially resolved analyses of immune‐stromal interactions and better mimics the stromal organization and diffusion gradients of the bone marrow TME compared to conventional 2D systems.^16^, ^26^, ^28^ Previous work by Lindacher et al. confirmed the structural and functional relevance of the model, showing hypoxia, active proliferation, and limited apoptosis of both BMSCs and malignant B cells in the scaffold core, indicative of protective stromal niches. Importantly, fluorescent dextran penetration demonstrated preserved drug diffusion, excluding impaired accessibility as a confounding factor.^26^ These features support the utility of the model to investigate mechanisms of immune resistance in spatially organized, disease‐specific TME contexts. Applying this system to patient samples, we demonstrated striking differences in CAR T‐cell function between CLL and ALL. These differences were generally most pronounced in the scaffold core regions and highlight the interplay between intrinsic T‐cell fitness and extrinsic suppression by the local microenvironment.

One prominent distinction was the differential expansion and spatial localization of CD8⁺ CAR T cells. ALL‐derived CAR T cells showed a marked post‐transductional increase in CD8⁺ T cells and reduced PD‐1 expression, aligning with their known proliferative advantage and superior clinical persistence.^34^, ^35^ In contrast, CLL‐derived CAR T cells retained a CD4‐dominant profile suggesting limited CD8⁺ expansion, as also described by Stock et al.^36^ In addition, the diminished cytotoxicity and elevated expression of exhaustion markers, particularly in scaffold core regions, extend prior reports of T‐cell dysfunction in CLL,^12^, ^37^, ^38^, ^39^, ^40^, ^41^ and suggest that both intrinsic and spatially organized extrinsic factors of the TME contribute to therapeutic failure. Following CAR transduction and 3D co‐culture, CLL‐derived CAR T cells retained a differentiated profile, with enrichment of effector memory (Tem) and central memory (Tcm) subsets, with markedly reduced frequencies of naïve and stem‐cell memory (Tscm) subsets. In contrast, ALL‐derived CAR T cells displayed higher proportions of early memory‐like phenotypes, particularly within the CD8^+^ subset, which are associated with long‐term persistence and durable antitumor activity.^9^, ^29^

Beyond impaired spatial engagement, CLL‐derived CAR T cells exhibited features of functional exhaustion shown by reduced proliferation, diminished secretion of cytotoxic mediators including granzyme A and perforin, and elevated expression of PD‐1, TIM‐3, and LAG‐3. These findings are consistent with reports of senescent, hypofunctional T cells in CLL^12^, ^37^, ^39^, ^40^, ^41^, ^42^, ^43^, ^44^ and align with clinical observations linking exhaustion marker expression to poor CAR T‐cell responses.^9^, ^45^ Notably, these defects were most pronounced in core regions of the 3D scaffold, suggesting that microenvironmental signals further enhance intrinsic dysfunction, which aligns with prior work showing that 3D architecture amplifies therapeutic resistance in core tumor regions.^28^ This spatial amplification of exhaustion phenotypes highlights the added value of our model for capturing clinically relevant resistance patterns. In summary, both phenotypic and spatial parameters converge to restrict the therapeutic fitness of CLL‐derived CAR T cells.

Acalabrutinib, Idelalisib, and Venetoclax are known to disrupt key survival and signaling pathways in CLL cells, limit TME‐mediated pro‐survival signals, and sensitize CLL cells to immune‐mediated clearance.^46^, ^47^, ^48^, ^49^, ^50^, ^51^, ^52^, ^53^, ^54^, ^55^, ^56^, ^57^ Combination therapies reduced exhaustion marker expression of CLL CAR T cells, in both peripheral and core regions, suggesting a potential improvement in CAR T‐cell functionality either indirectly by depletion of CLL cells or through direct interactions. Among the tested compounds, BTK inhibition with Acalabrutinib enhanced tumor cell depletion and reduced exhaustion marker expression in the core regions, consistent with previous observations, which improved CAR T‐cell expansion and reduced dysfunctional subsets, including exhausted T cells and regulatory T cells (Tregs).^46^, ^48^, ^49^, ^50^, ^52^, ^58^ Our findings therefore support a role of BTK inhibition in reversing CAR T‐cell exhaustion, particularly in stromal‐protected tumor regions.

PI3Kδ blockade with Idelalisib likewise increased tumor cell depletion and decreased exhaustion in our setting, in line with its reported ability to modulate T‐cell differentiation, enrich for less differentiated cytotoxic CD8⁺ subsets, reduce Tregs, and lower exhaustion marker expression.^36^, ^53^ However, Idelalisib has been associated with immune‐mediated toxicities in CLL patients, likely due to its disproportionate impact on Tregs.^59^, ^60^ Since Tregs contribute to the immunosuppressive TME in CLL, selective modulation of this balance may enhance antitumor immunity and improve CAR T‐cell efficacy.^53^, ^54^, ^60^, ^61^ Given the risk of impairing CAR T‐cell functionality, optimal timing and dosage of Idelalisib is crucial. Our data suggest that pre‐treatment or short‐term co‐treatment with Idelalisib may provide functional benefits by reducing CAR T‐cell exhaustion while preserving cytotoxic activity.

Venetoclax, a selective Bcl‐2 inhibitor, further reduced exhaustion without impairing CAR T‐cell viability, likely through enhanced target cell elimination rather than direct modulation of CAR T‐cell biology, supporting current trials combining Venetoclax with CAR T therapy in CLL (NCT03331198). Previous studies reported enhanced CAR T‐cell cytotoxicity upon Venetoclax treatment, particularly following tumor cell pre‐sensitization, while prolonged high‐dose exposure induced dose‐dependent CAR T‐cell apoptosis.^55^, ^56^ Venetoclax treatment has been associated with a reduction of Tregs and exhausted T cells with recovery of T‐cell function in CLL.^57^ Notably, low‐dose Venetoclax in our setting did not impair CAR T‐cell survival.

Targeting IL‐10 under 3D conditions enhanced cytotoxicity and reduced exhaustion of CLL‐derived CAR T cells, particularly in the core regions, which aligns with preclinical CLL models where IL‐10 blockade reduced leukemic burden and enhanced T‐cell‐mediated antitumor responses,^17^, ^33^ as well as observations in other tumor types.^32^, ^62^, ^63^ In CLL, elevated IL‐10 levels contribute to an immunosuppressive TME and enhanced CD8⁺ T‐cell exhaustion, which can be partially reversed by IL‐10 blockade.^64^ However, IL‐10 is not solely suppressive. Hanna et al. demonstrated that IL‐10R–STAT3 signaling preserves progenitor‐like CD8⁺ T‐cell subsets and prevents premature exhaustion, highlighting the dual role of IL‐10. Tumor‐derived IL‐10 promotes immunosuppression, whereas IL‐10R signaling can sustain functional memory‐like subsets.^65^ For CLL, this suggests that transient IL‐10 blockade may counteract tumor‐driven suppression, while preserving long‐term IL‐10R signaling required to maintain progenitor‐like CD8⁺ T cells. Our findings therefore support a model in which short‐term IL‐10 inhibition, administered prior to and during early CAR T‐cell activity, could disrupt TME‐driven immunosuppression without incurring the risks of sustained cytokine inhibition. Of note, Tregs were not removed during T‐cell enrichment and are therefore likely present in our CAR T‐cell products. Given their abundance in CLL and capacity to secrete IL‐10 and suppress effector responses, Tregs may further contribute to the immunosuppressive environment observed in our model, consistent with recent reports highlighting the potential benefit of Treg depletion strategies to improve CAR T‐cell efficacy in CLL.^66^ In addition, exogenous IL‐2 supplementation has recently been reported to restore CAR T‐cell activation in CLL,^67^ suggesting that cytokine support may represent an additional strategy to enhance CAR T‐cell function alongside the pharmacological and TME‐targeted interventions explored here.

Additionally, we explored the CXCR4/CXCL12 signaling axis, a key mediator of CLL retention in stromal niches and driver of therapy resistance.^23^, ^25^, ^68^, ^69^ Consistent with prior data linking CXCL12 to IL‐10 production via STAT3,^20^ our model showed concurrent upregulation of IL‐10 and CXCR4 in core‐localized CLL cells,^26^ suggesting a spatially confined immunosuppressive signaling loop. CXCR4 enrichment in the core‐localized CLL cells likely reflects a dynamic and context‐dependent regulation. CXCR4 undergoes rapid internalization upon ligand engagement, suggesting that surface levels fluctuate with the timing and intensity of stromal interactions. Moreover, CXCR4 is transcriptionally regulated by hypoxia through HIF‐1α, which is more pronounced in the scaffold core, providing a mechanistic explanation for the enrichment of CXCR4⁺ CLL cells in this region.^47^, ^70^ That aligns with transcriptomic analysis, which demonstrated concurrent upregulation of IL‐10 and CXCR4 in core‐resident CLL cells.^26^ Taken together, these data support a model in which hypoxia and stromal protection synergize to sustain CXCR4 expression, thereby promoting leukemic cell retention and survival. Importantly, pharmacologic blockade with Motixafortide prevented CXCR4‐mediated niche retention,^25^, ^71^, ^72^ when applied at the time of leukemic cell addition, leading to enhanced CAR T‐cell cytotoxicity in core regions. In parallel, CXCR4 inhibition reduced exhaustion marker expression in CAR T cells in the core regions, consistent with reports that CXCR4 signaling contributes to dysfunction via the JAK2/STAT3–TOX pathway.^73^ These findings highlight a dual benefit of CXCR4 inhibition on CAR T‐cell fitness, disrupting spatial retention of malignant cells and relieving T‐cell exhaustion.^9^, ^73^

Our 3D co‐culture system recapitulates key structural and functional features of the CLL bone marrow microenvironment, including spatial compartmentalization, hypoxia, and stromal support. By enabling spatially resolved readouts and maintaining autologous cell–cell interactions, the model captures critical resistance mechanisms that are absent in conventional 2D systems and provides a relevant platform for dissecting tumor‐immune interactions and testing targeted combination strategies. Nonetheless, it remains an in vitro system and lacks systemic immune interactions present in vivo. In particular, our platform does not include myeloid populations, such as macrophages or dendritic cells, which are abundant in the CLL TME and critically shape immune responses. Furthermore, the scaffold composition mimics bone marrow stiffness but may not fully represent other CLL‐involved tissues, such as lymph nodes or spleen. Therefore, further studies are needed to validate our findings in clinical settings. Future settings employing bulk RNA‐sequencing of CLL‐ and ALL‐derived CAR T cells will be essential to dissect signaling differences at the transcriptional level, representing the next step to mechanistically define disease‐specific CAR T‐cell dysfunction.

In conclusion, our data suggest that CAR T‐cell dysfunction in CLL is driven by a combination of intrinsic T‐cell impairments and extrinsic immunosuppressive signals from the TME. Targeting these factors through combinatorial strategies, such as blockade of IL‐10 or CXCR4, may enhance CAR T‐cell efficacy and overcome therapeutic resistance in CLL patients.

## MATERIAL AND METHODS

### Primary cellular material and cell lines

Peripheral blood was obtained from patients with CLL and ALL. All patients gave written informed consent according to CARE guidelines and in compliance with the Declaration of Helsinki. PBMCs were isolated from fresh blood by Ficoll‐Hypaque density gradient centrifugation and cryopreserved. Detailed patient characteristics are provided in Tables S1 and S2. Autologous CD4^+^ and CD8^+^ T cells were co‐enriched from cryopreserved patient‐derived PBMCs using CD4 and CD8 microbeads (Miltenyi Biotec, Bergisch Gladbach, Germany) according to manufacturer instructions and used for CAR T‐cell generation. No additional depletion of T‐cell subsets, including Tregs, was performed, and thus, Tregs are likely present in the CAR T‐cell products. The remaining PBMCs, including malignant B cells, were cryopreserved until co‐culture. In addition, T and B cells were enriched from cryopreserved PBMCs of HDs to generate CAR T cells for 2D functionality analysis. Primary CLL and ALL cells were cultured in RPMI 1640 medium (Gibco, Waltham, Massachusetts, USA), supplemented with 10% fetal calf serum (FCS, ccpro, Oberdorla, Germany), 1% l‐glutamine (Thermo Fisher Scientific, Waltham, Massachusetts, USA), 0.6% MEM NEAA (PAN Biotech, Aidenbach, Germany), 1% penicillin/streptomycin, 1% HEPES, 1% Na‐pyruvate, 0.2% antibiotic‐antimycotic, and 0.007% β‐mercaptoethanol (Gibco). Primary T cells were cultured in TexMACS medium (Miltenyi Biotec), supplemented with IL‐7, IL‐15 (Miltenyi Biotec) at a concentration of 12.5 µg/L and 3% human AB serum (ccpro, Germany). The BMSC human cell line HS‐5 (CRL‐11882, ATCC, Manassas, USA) was cultured in DMEM + GlutaMAX (Gibco) supplemented with 10% FCS and 0.4% penicillin/streptomycin. CD19^+^ Nalm‐6 (ACC 128, DSMZ, Braunschweig, Germany), CD19^+^ Mec‐1 (ACC 497, DSMZ), and CD19^–^ U937 cells (ACC 5, DSMZ) were used as control cell lines for 2D functionality analysis and cultivated in medium for primary CLL cells (Mec‐1) or RPMI supplemented with 10% FCS and 0.4% penicillin/streptomycin (Nalm‐6, U937). HEK293T/17 cells (CRL‐11268, ATCC) were cultured in DMEM + GlutaMAX (Gibco) supplemented with 10% FCS for lentiviral vector production. All cell cultures were maintained at 37 °C in a fully humidified incubator with 5% CO₂ and routinely tested negative for mycoplasma contamination. The study was approved by the Ethics Committee of the University of Erlangen‐Nürnberg (number: 219_14B, addendum 59_17 Bc).

### Description of the chimeric antigen receptor vector

The lentiviral vector used to generate third‐generation CD19‐directed CAR T cells was kindly provided by the working group of Prof. Axel Schambach (Institute of Experimental Hematology, Hannover Medical School). The vector encodes a single‐chain variable fragment (scFv) derived from a mouse hybridoma FMC63, CD19‐targeting domain, hinge and transmembrane regions from the human CD8α molecule, a CD28 and 4‐1BB cytoplasmic costimulatory domain, and a cytoplasmic CD3ζ T‐cell activation domain (Figure 1A).

### Production of lentiviral supernatant

In order to produce lentiviral supernatant, HEK293T/17 cells were seeded (T175) and cultured for 24 h to reach approximately 80% confluency. Cells were transfected as follows: DNA solution was prepared by adding 70 µg of lentiviral transfer plasmid, 52.5 µg of psPAX2 packaging plasmid, and pCMV‐VSV‐G envelope plasmid to 8 mL Opti‐MEM medium (Invitrogen, Carlsbad, USA). The psPAX2 was a gift from Didier Trono (Addgene, Watertown, USA, plasmid #12260; http://n2t.net/addgene:12260; RRID:Addgene_12260). The pCMV‐VSV‐G was a gift from Bob Weinberg (Addgene plasmid #8454; http://n2t.net/addgene:8454; RRID:Addgene_8454).^74^ The lipofectamine mix consisted of 300 µL Lipofectamine^TM^ 2000 Reagent (Thermo Fisher Scientific) and 7.7 mL Opti‐MEM (Gibco), which was added to the DNA solution after 5 min of pre‐incubation at room temperature. The Lipofectamine‐DNA mixture was incubated for 15 min and added to HEK293T/17 cell flasks containing freshly changed medium. After 6 h, the medium was replaced with T‐cell medium. For control T‐cell approaches, a mock supernatant without a transfer plasmid was generated analogously to the lentiviral CAR vector supernatant. After 48 h, viral supernatants were collected, filtered through a 0.2 μM filter (Merck, Darmstadt, Germany), and cryopreserved at −80°C.

### CAR T‐cell generation

Autologous CD4^+^ and CD8^+^ T cells were seeded at a density of 5 × 10^5^ per 24‐well in T‐cell medium, supplemented as described above, and stimulated with TransAct (Miltenyi Biotec) according to the manufacturer's instructions. Of note, we did not supplement cultures with IL‐2, as IL‐7 and IL‐15 are known to better support Tcm and Tscm maintenance, which likely explains the relatively high proportion of these subsets observed in both CLL‐ and ALL‐derived CAR T cells. After 24 h, medium was removed, and lentiviral supernatant, mixed with 10 µg/mL Vectofusin‐1 (Miltenyi Biotec), was added at an MOI of 3. To enhance transduction efficiency, T cells were centrifuged at 700 × *g* for 45 min at 32°C and then transferred to the incubator. After 48 h, the viral supernatant was replaced with fresh T‐cell medium. Cells were expanded for 12–14 days, harvested, and analyzed for CAR expression, followed by cryopreservation.

### Flow cytometry

For flow cytometry preparation, cells were washed with phosphate‐buffered saline (PBS) and stained with the appropriate antibodies and reagents (Table S3), which were diluted in staining buffer (0.5% bovine serum albumin, 2.5 mM ethylenediaminetetraacetic acid in PBS). The staining was performed at room temperature, protected from light, for 10 min. For intracellular staining, the Fix & Perm^TM^ Cell Permeabilization Kit (Invitrogen) was used according to the manufacturer's instructions. Sample data were acquired on the BD FACSCanto^TM^ II Flow Cytometry System (BD Biosciences, San Jose, California). Data analysis was performed using the Kaluza Software 2.1 (Beckman Coulter, Brea, California).

### 2D functionality assays

HD‐derived CAR T cells and mock‐transduced control T cells were co‐cultured with donor‐matched B cells, CD19^+^ Mec‐1 cells, CD19^+^ Nalm‐6 cells, and CD19^–^ U937 cells as a negative control at an E:T ratio 1:1. Cells were co‐cultured in equal parts of TexMACS T‐cell medium and CLL/ALL medium.

For activation and cytotoxicity assay, cells were co‐cultured for 18 h. After incubation, cells were analyzed by flow cytometry. Target cells and T cells were differentiated by CD19/CD3 staining. The percentage of living target cells was determined by 7‐AAD^–^ CD3^–^ and used to calculate cytolysis by the following equation: % cytolysis = (% live cells [target cells alone] − % live cells [sample of interest])/% live cells (target cells alone) × 100.

For the degranulation assay, mock‐transduced T cells and CAR T cells were left unstimulated as a negative control or stimulated with 50 ng/mL PMA (Sigma‐Aldrich, St. Louis, Missouri, USA) and 0.1 µM Ionomycin (Cayman, Ann Arbor, Michigan, USA) as a positive control or co‐cultured with target cells. Directly after seeding, CD107a‐BV421 antibody (1:25, Biolegend, San Diego, California, USA) or the corresponding isotype control was added to the culture. After 1 h of incubation, Golgi‐Stop^TM^ Protein Transport inhibitor (BD Biosciences) was applied (1:1000). After 6 h, cells were analyzed by flow cytometry.

For the proliferation assay, CAR T cells and mock‐transduced T cells were stained with Cytopainter (CP) Cell Proliferation Staining Reagent (Abcam, ab176736, Cambridge, UK) according to the manufacturer's protocol prior to seeding. Flow cytometry was performed directly after seeding and on Day 4. The CP^Dim^ gate was based on the mock‐transduced T‐cell monoculture on Day 4 as a reference. A gating strategy illustrating the proliferation of CAR T cells is shown in Figure S1.

For live cell cytotoxicity analysis, primary CLL cells were stained with 0.75 mM Incucyte Cytolight Dye Green (Sartorius, Göttingen, Germany) and added to confluent HS‐5 cells at a concentration of 50 × 10^4^ cells/96‐well. CAR T cells or mock‐transduced T cells were added to the co‐culture at an E:T ratio of 1:1. After Incucyte Annexin Red Dye (Sartorius) was applied directly into the medium, the co‐cultures were imaged every 30 min in green (excitation 440–480 nm; emission 504–544 nm) and red (excitation 565–605 nm; emission 625–705 nm) fluorescent channels for up to 36 h. For image calibration, the area assessment was used (IncuCyte SX1). As a measure of cell apoptosis, the area (µm^2^/image) of double‐positive CLL cells (Cytolight green^+^/Annexin red^+^) was automatically quantified by IncuCyte Base Analysis Software 2022A (Sartorius). Cells ≤ 10 µM were excluded as background. Data were corrected by subtraction of spontaneous, basal lysis and normalized to the maximal lysis signal of CAR T cells in the assay.

### 3D cell culture

For 3D co‐cultivation, highly porous polystyrene Alvetex® scaffolds (Reprocell Europe, Durham, UK) were used, with a pore size of 36–40 µm, a surface area of 1.9 cm², and a thickness of 200 µm. Scaffolds were prepared according to the manufacturer's protocol and additionally coated with 0.1% gelatin for 20 min. HS‐5 BMSC cells were added to the center of the scaffold in a volume of 70 µL and incubated for 1 h before the medium was applied. After 7 days, cryopreserved primary CLL or ALL cells were added in a total number of 8 × 10⁵ malignant B cells. The culture was incubated for an additional 4 days. Patient‐derived autologous CAR T cells or mock‐transduced control T cells, which were thawed and rested for 2 h prior to seeding, were applied at an E:T ratio of 1:1 in co‐culture medium consisting of 75% HS‐5‐medium, 12.5% CLL medium, and 12.5% TexMACs medium. Cells were recovered separately from the periphery and the core of the scaffold. Peripheral cells were defined as loosely adherent populations that could be removed by rinsing the scaffolds in a circular motion multiple times and collecting the supernatant. Cells that remained embedded within stromal‐dense inner core regions required mechanical disruption of the scaffold for recovery. The scaffolds were sliced into small pieces and incubated in PBS at 37°C, 250 rpm for 15 min by agitation.

### 3D cytotoxicity assay

For the 3D cytotoxicity assay, autologous mock‐transduced T cells or CAR T cells were added to CLL‐ or ALL‐derived co‐cultures and incubated for 18 h. After incubation, the cells were stained and analyzed by flow cytometry. B and T cells were differentiated by CD19/CD3 staining. The percentage of live CD19^+^ malignant B cells was determined by Zombie NIR^TM^ Fixable Viability Dye (Biolegend) staining and used to calculate the cytolytic activity of the effector T cells using the same equation used in the 2D cytotoxicity assay above. A representative gating strategy is shown in Figure S2.

### Immunofluorescence staining

After 18 h of co‐culturing with mock‐transduced T cells or CAR T cells derived from CLL or ALL patients, the scaffold was removed from the cell culture plate, cut into pieces, and transferred to an 8‐well chamber slide (IBIDI, Gräfelfing, Germany). The scaffold pieces were washed twice with PBS, fixed with 4% paraformaldehyde for 10 min, and permeabilized with 0.1% Triton‐X100 for 15 min at room temperature. In order to avoid unspecific binding, cells were incubated in blocking buffer for 2 h and stained with primary antibodies (Table S3) overnight at 4°C. Following additional washing steps, staining with secondary antibodies was performed for 2 h at room temperature. Nuclei were counterstained with NucGreen (Invitrogen). The 3D cultures were mounted with ProLong Glass Antifade Mountant (Invitrogen), dried overnight, and stored at 4°C. Confocal laser imaging was performed on a Leica Stellaris 8 (Wetzlar, Germany) using Fiji with the 3Dscript plugin for visualization.^75^, ^76^

### Image analysis and quantification

To segment T and B cells, all raw *z*‐stacks capturing different views of the same scaffold were initially processed in Fiji using morphological filters,^75^ followed by applying a 3D variance filter with a radius of two pixels in every dimension. Random slices along the *x*–*y* dimensions were manually annotated in Cellpose (v3.0.7),^77^ yielding 1455 distinct cell masks from the NucGreen channel. These masks were employed to train a Cellpose model, starting from the pre‐trained nuclei model with a learning rate of 0.1, a weight decay of 0.0001, 500 epochs, a cell probability of 1.5, and an estimated diameter of 10.2 pixels. Training was performed in Python (v3.8.18) using an NVIDIA T400 4GB GPU. Cells were subsequently segmented via Cellpose using the trained model, with an estimated diameter of 10.2 pixels.

The segmented cell masks were analyzed with Python (v3.11.5) and the Nyxus package (v0.7.5) to measure CD3 and CD19 intensities for cell classification and counting. The total pixel intensity of CD3 and CD19 for each cell mask per *z*‐stack was calculated, min–max normalized, and log‐transformed. The intensity distributions were aggregated per patient to determine threshold values for CD19^+^ and CD3+ cells based on FACS measurements that were averaged across the corresponding cell cultures for either ALL or CLL co‐cultures, respectively. Mean fractions of CD3^+^ or CD19^+^ cells across patient‐derived samples for either ALL or CLL samples under both experimental conditions (+mock‐transduced T cells/+CAR T cells) were evaluated, and the threshold was set at the *n*th percentile with *n* = 100–mean (fraction of CD3^+^/CD19^+^ cells). These thresholds were used to classify and count the CD19^+^ and CD3^+^ cells in each *z*‐stack. Cell masks that were positively marked for both CD19^+^ and CD3^+^ were classified as co‐localized.

### Cytokine release

To analyze the release of cytokines and chemokines from 3D co‐cultures, supernatants from autologous CLL‐ and ALL‐derived co‐cultures were collected after 24 h of co‐cultivation with CAR T cells or mock‐transduced T cells and stored at −80°C. Cytokines were detected using multiplex immunoassay LEGENDplex^TM^ Human CD8/NK Panel (Biolegend, 741 187), while chemokine detection was performed using LEGENDplex^TM^ HU Proinflammatory Chemokine Panel 1 (Biolegend, 740 985), following the manufacturer's instructions. For quantification of cytokine/chemokine release, the LEGENDplex^TM^ analysis software (Biolegend) was used. All LEGENDplex analytes not shown in the results are provided in Figure S3. IL‐10 release was additionally quantified by ELISA (Biolegend). Successful neutralization of IL‐10 was validated by ELISA using the neutralizing IL‐10 antibody as capture antibody (Figure S4C).

### Inhibitor assay

For the inhibitor assay, primary CLL cells were stained with CP Cell Proliferation Staining Reagent (Abcam) prior to adding to HS‐5‐confluent scaffolds. After 3 days of stromal contact, the co‐cultures were treated with Acalabrutinib (Cayman Chemical, Michigan, USA), Idelalisib (Cayman), or ABT199 (Cayman) for 72 h in total. After 48 h of treatment, autologous mock‐transduced T cells or CAR T cells were added for an additional 24 h in the presence of inhibitors. In the case of treatment with the neutralizing IL‐10 antibody (Biolegend, clone JES3‐9D7) or CXCR4 antagonist Motixafortide (MTF, Hycultec, Passau, Germany), the inhibitors were administered to the co‐culture immediately after seeding the CP‐stained primary CLL cells. After 72 h of treatment, autologous mock‐transduced T cells or CAR T cells were applied and incubated for an additional 24 h in the presence of anti‐IL‐10 or MTF. All inhibitors were re‐added every 48 h except for ABT199. Dimethyl sulfoxide (DMSO) was used as a solvent control. For IL‐10 neutralization, an isotype control antibody was included in a subset of samples from three CLL patients to exclude non‐specific or toxic effects of the antibody itself (Figure S4B). After treatment, cells were harvested from the peripheral and the core regions of the scaffolds, and flow cytometric analysis was performed. To assess CLL survival, cells were stained with Annexin V (Biolegend) and 7‐AAD (BD Biosciences) in Annexin V Binding Buffer (BD Biosciences). Additionally, the exhaustion of effector cells was determined by TIM‐3, LAG‐3, and PD‐1 surface staining.

### Statistical analysis

Data processing and graphical representation were performed with GraphPad Prism v9.5.1 (GraphPad Prism Software Inc., San Diego, USA). Depending on the data set, statistical significance was determined by a two‐tailed unpaired or paired *t*‐test, Mann–Whitney *U* test, two‐way analysis of variance (ANOVA) with Šidák´s multiple comparisons test, or Tukey's multiple comparisons test (one‐way or two‐way ANOVA). Differences were considered significant if P ˂ 0.05.

## AUTHOR CONTRIBUTIONS

**Janin Dingfelder**: Writing—original draft; investigation; methodology; visualization; software; formal analysis; data curation. **Michael Aigner**: Conceptualization; writing—review and editing. **Jana Lindacher**: Methodology; writing—review and editing. **Pascal Lukas**: Software; formal analysis; writing—review and editing. **Cindy Flamann**: Methodology; formal analysis. **Gina Nusser**: Methodology; formal analysis. **Katharina Zimmermann**: Resources; writing—review and editing. **Axel Schambach**: Resources; writing—review and editing. **Frederik Graw**: Formal analysis; software; writing—review and editing. **Simon Völkl**: Resources; writing—review and editing. **Heiko Bruns**: Resources; writing—review and editing. **Manuela Krumbholz**: Conceptualization. **Martina Haibach**: Resources; writing—review and editing. **Jochen Wilke**: Resources. **Andreas Mackensen**: Conceptualization; writing—review and editing. **Gloria Lutzny‐Geier**: Conceptualization; supervision; funding acquisition; writing—original draft; formal analysis; data curation; project administration.

## CONFLICT OF INTEREST STATEMENT

The authors declare no conflicts of interest.

## ETHICS STATEMENT

All procedures were conducted in accordance with the Good Clinical Practice guidelines of the International Council for Harmonization and covered by license number: 219_14B, addendum 59_17 Bc. All participants gave written informed consent according to CARE guidelines and in compliance with the Declaration of Helsinki and 24‐487‐Bp_2024.12.19.

## FUNDING

J.D. was supported in part by the Wilhelm‐Sander Foundation (2020.045.1). G.L.‐G. was supported by the Deutsche Forschungsgemeinschaft (DFG‐LU 2181/1‐2) and GILEAD (PO 201035124). J.L. was supported by the Wilhelm‐Sander Foundation (2021.059.1). F.G. and P.L. were supported by the German Federal Ministry of Science and Education (BMBF, 031L0293E to F.G.) and the Hightech Agenda Bavaria (F.G.). Cell collection and immunofluorescence analyses have been performed at the Core Units “Cell Sorting and Immunomonitoring,” and the “Optical Imaging Center,” Erlangen. Open Access funding enabled and organized by Projekt DEAL.

## Supporting information

Supplementary Information

## Data Availability

The data that support the findings of this study are available from the corresponding author upon reasonable request.
