# Supplementary material for: Functional differences between CLL‐ and ALL‐derived CAR T cells in a 3D tumor microenvironment highlight CXCR4 and IL‐10 as potential modulatory targets
Source: Hemasphere. 2025 Dec 8;9(12):e70279. doi: 10.1002/hem3.70279 (PMC12683941; doi:10.1002/hem3.70279)
Supplement: Supplementary file 1 — Supplementary Information [file HEM3-9-e70279-s001.docx]

**Supplement**

^
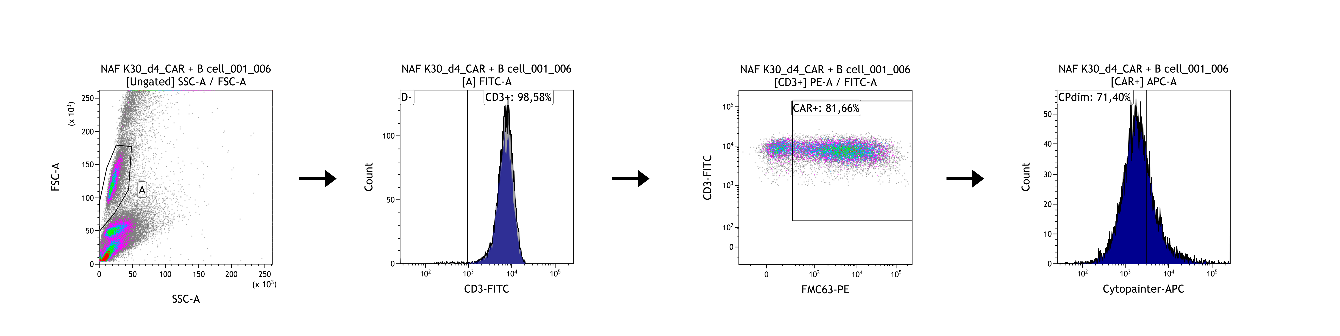
^

**Supplement Figure S1: Gating strategy illustrating proliferation of patient-derived CAR T-cells**. Representative flow cytometric plots of Cytopainter Deep Red dilution to track T-cell proliferation at day 4 after target cell contact. Cell debris was excluded by FSC-A/SSC-A. CAR T-cells were gated by CD3^+^ CAR^+^ and proliferating CAR T-cells were gated by Cytopainter Deep Red dilution (CP Dim).


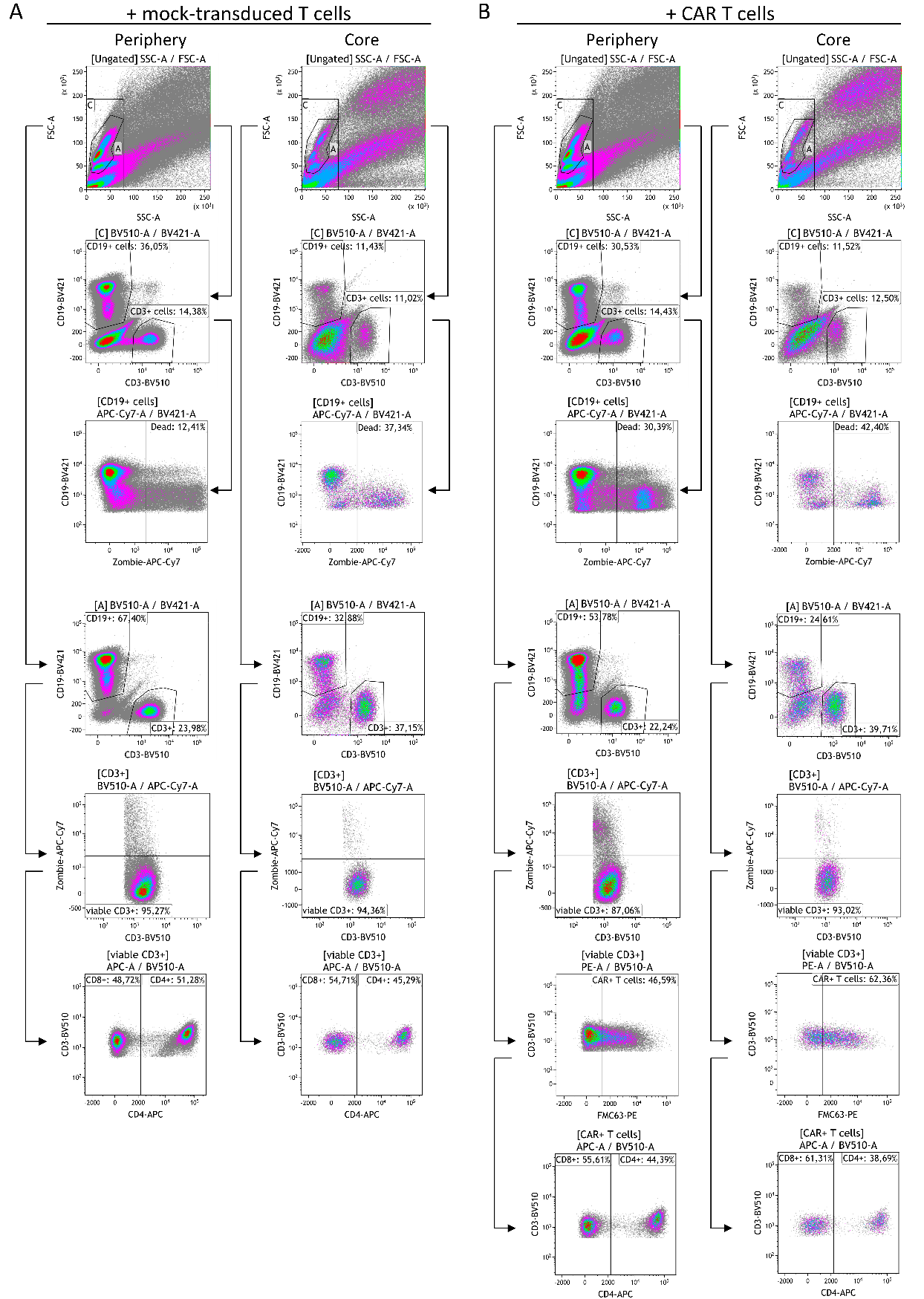


**Supplement Figure S2: Gating strategy demonstrating cellular distribution within the 3D model and cytotoxicity of patient-derived CAR T-cells.** Representative flow cytometric plots of 3D co-culture with CLL-patient derived B cells and (A) mock-transduced T-cell and (B) CAR T-cells. For cytotoxicity analysis a major part of HS-5 stromal cells was excluded by FSC-A/SSC-A and the remaining cells subdivided into CD19^+^ B cells and CD3^+^ T-cells for the periphery and core regions. B cells were subdivided in live cells by Zombie Dye^−^ CD19^+^ and dead cells by Zombie Dye^+^ CD19^+^. To determine the cellular distribution within the co-culture in peripheral and core regions, the populations of interest were pre-gated with the exclusion of cell debris using FSC-A/SSC-A and then subdivided into CD19^+^ B cells and CD3^+^ T-cells. Viable CD3^+^ T-cells were identified by CD3^+^/Zombie dye^-^ gating and further subdivided into viable CD3^+^ CAR^+^ T-cells in case of CAR T-cell co-cultures. The viable CD3^+^ T-cells or CAR T-cells were then further subdivided into CD4^+^ and CD8^+^ T-cells.


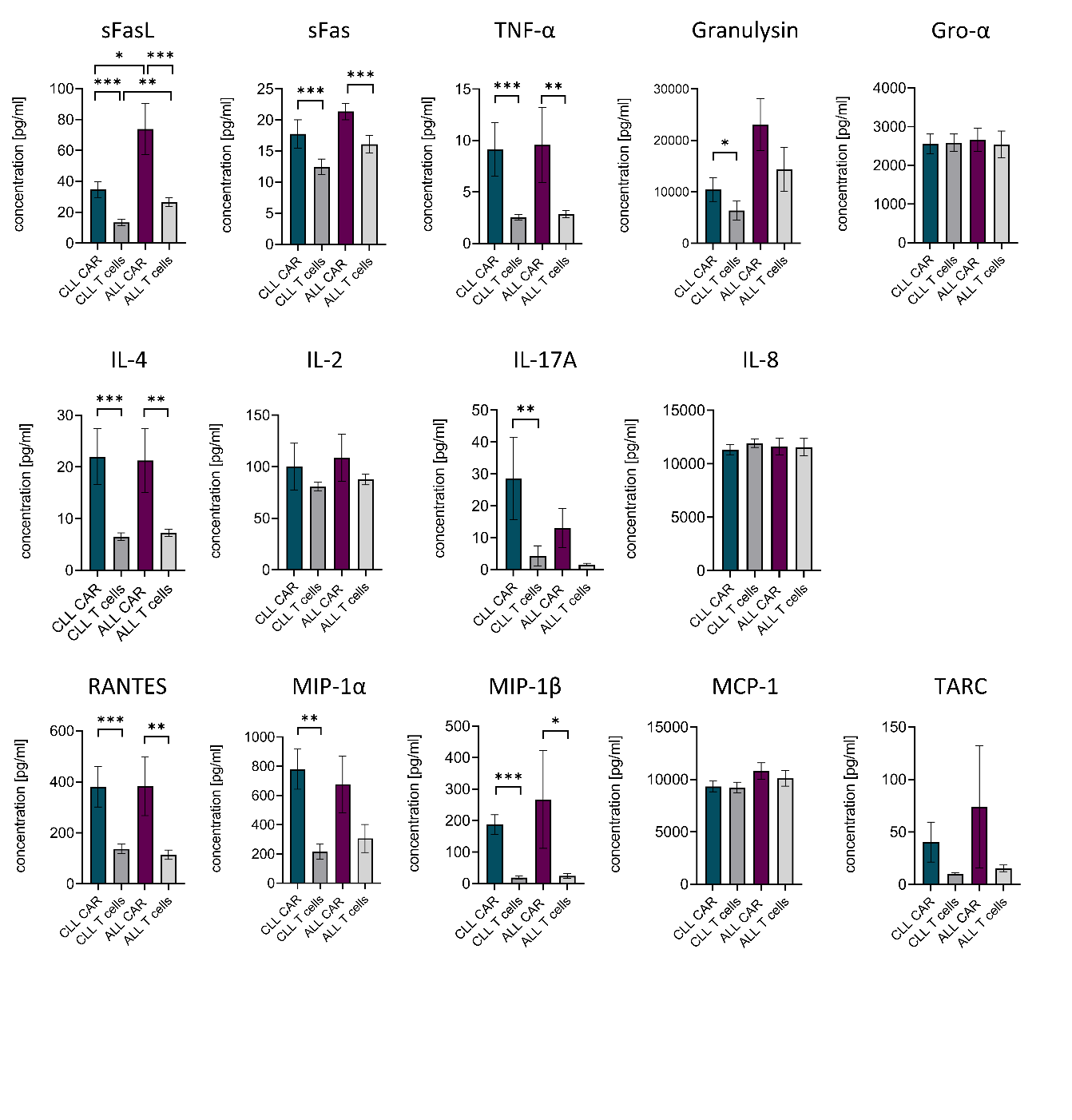


**Supplement Figure S3: Remaining cytokines and chemokines measured by multiplex immunoassay.** Release of cytokines and chemokines not included in main figures after 24 hours of 3D co-culture with chronic lymphocytic leukemia (CLL)- or acute lymphoblastic leukemia (ALL)-derived mock-transduced T-cells or CAR T-cells, measured by multiplex immunoassay of cell culture supernatants. Data are presented as mean ± SEM (n=8). Analytes with values outside the standard detection range were excluded. sFasL: soluble Fas ligand; sFas: soluble Fas; TNF-α: Tumor necrosis factor alpha; Gro-α: Growth-regulated alpha protein; IL: Interleukin; RANTES: Regulated upon activation, normal T-cell expressed and secreted; MIP: Macrophage inflammatory protein; MCP-1: Monocyte chemotactic protein 1; TARC: Thymus and activation-regulated chemokine.


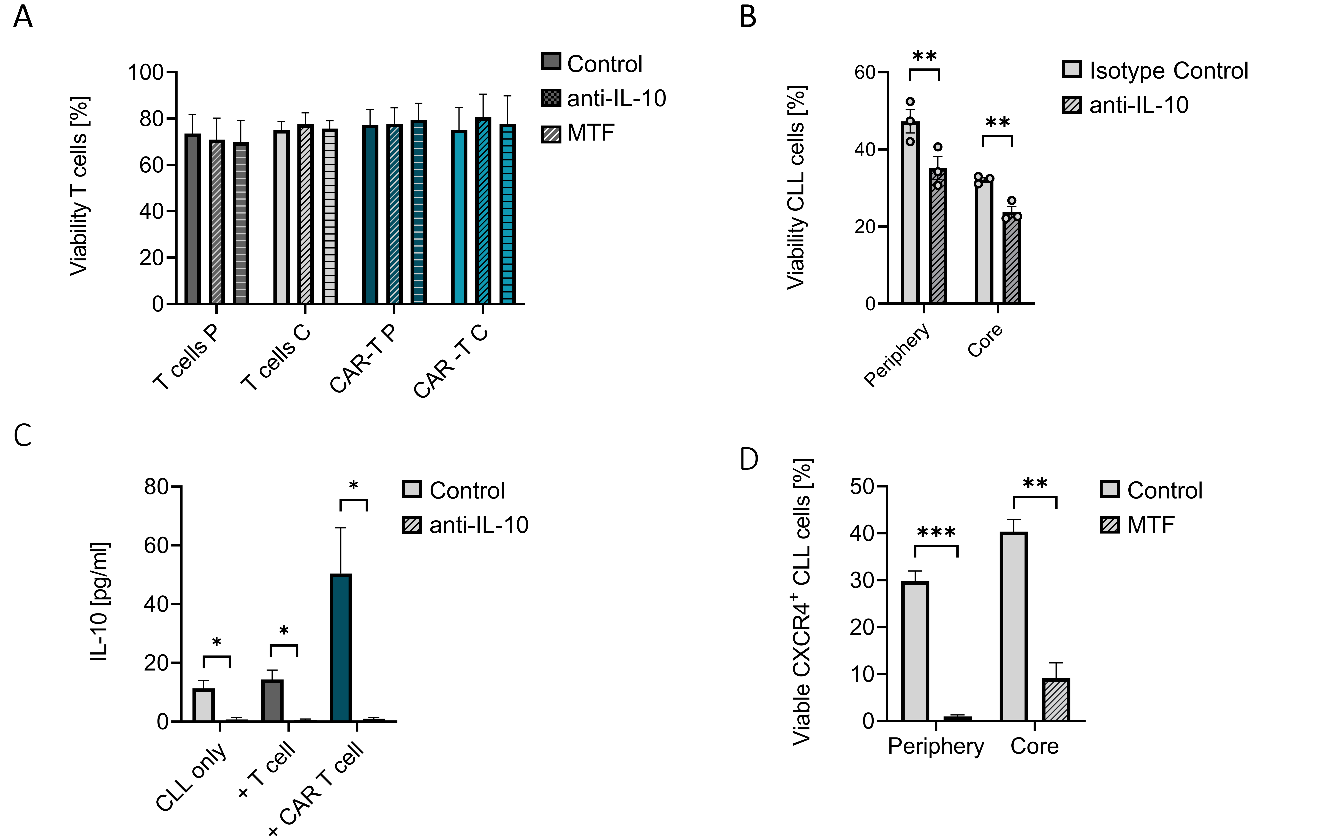
 **Supplement Figure S4: Viability of CLL-derived T-cells and CAR T-cells after treatment with anti-IL-10 antibody or MTF and validation of successful IL-10 and CXCR4 blockade.** (A) Viability of CLL-derived T-cells and CAR T-cells after treatment with anti-IL-10 antibody or Motixafortide (MTF) assessed by Annexin V/7-AAD staining (n=4). Data were analyzed by Dunnett’s multiple comparison test (Two-way ANOVA). (B) Viability of CLL cells after treatment of CLL/HS-5 3D co-culture (n=3) for 96 h with isotype control antibody and anti-IL-10 antibody to exclude non-specific or toxic effects of the IL-10 antibody itself. Data were analyzed by paired t-test. (C) Validation of full IL-10 blockade through anti-IL-10 antibody treatment in co-culture approaches depicted in Figure 7A measured by ELISA. (D) Blockade of CXCR4 on viable CLL cells through MTF assessed by flow cytometric analysis of CXCR4 surface expression in presence of CAR T-cells (n=4). Data were analyzed by paired t-test (C, D). Bars are represented as mean ± SEM. Significance is indicated by p* = 0.05; p** = 0.01; p*** = 0.001.


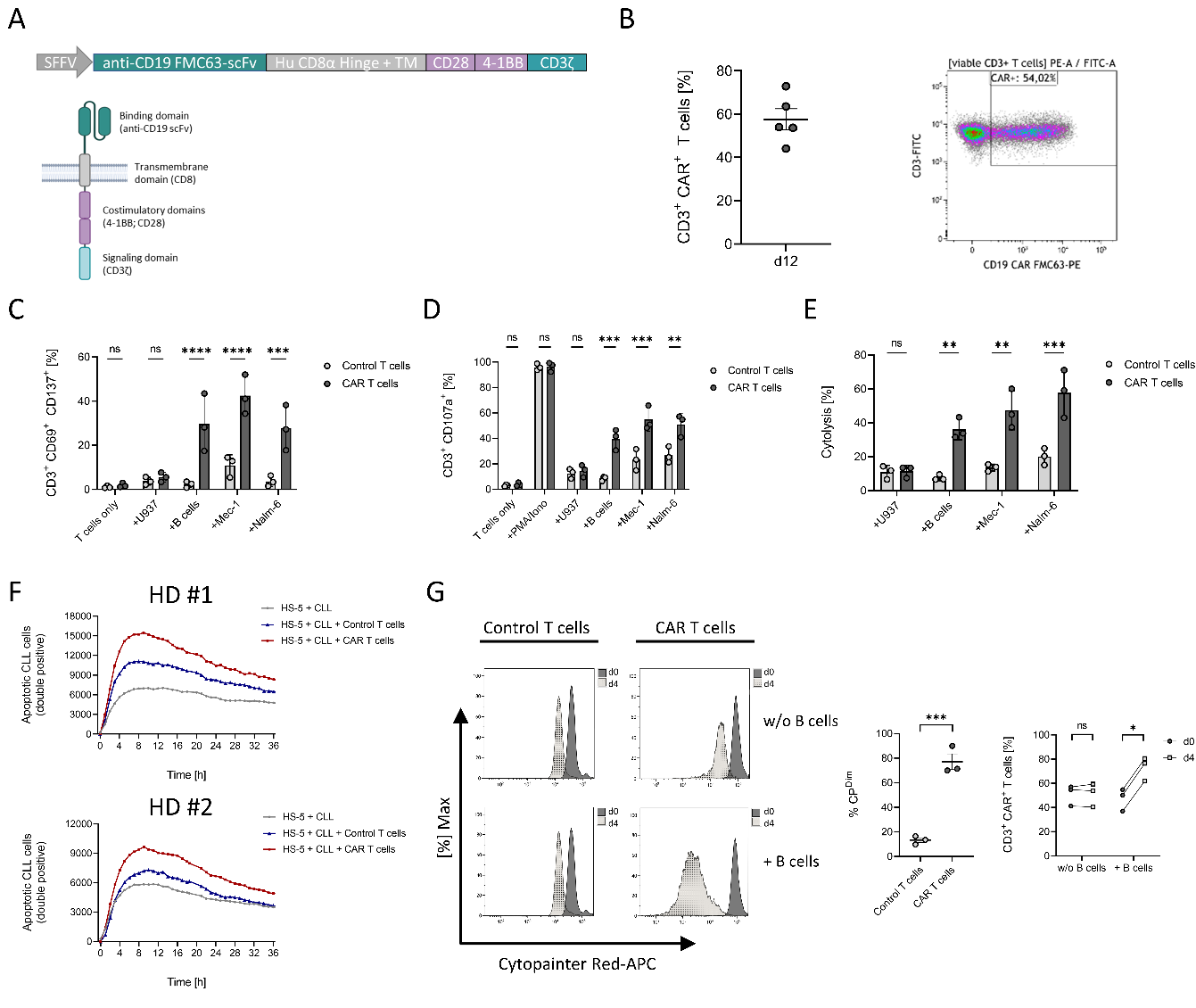
**Supplement Figure S5. Functionality of healthy donor derived CD19 CAR T-cells in 2D co-culture.** (A) Composition of the lentiviral CD19 CAR vector encoding a mouse hybridoma-derived FMC63 single-chain variable fragment (scFv), CD19-targeting domain, a human CD8α hinge and transmembrane domain, CD28 and 4-1BB cytoplasmic costimulatory domains, and a cytoplasmic CD3ζ cytoplasmic domain. (B) Transduction efficiency of five healthy donor (HD) derived CAR T-cells assessed at day 12. Data are shown as individual values with indication of the mean. A representative example of CAR^+^ gating is shown on the right. (C) Percentage of early antigen-specific activated CD69^+^ and CD137^+^ mock-transduced T-cells and CAR T-cells (HD, n=3) after 18 hours of co-culture with CD19^−^ U937 cells as negative control, donor-matched autologous B-cells, CD19^+^ Mec-1 cells or CD19^+^ Nalm-6 cells at an Effector:Target (E:T) ratio of 1:1. (D) Degranulation of mock-transduced T-cells and CAR T-cells (HD, n=3) following stimulation with PMA (50ng/ml) and Ionomycin (0.1µM) or co-culture with CD19^−^ U937 cells, donor-matched autologous B-cells, CD19^+^ Mec‑1 cells or CD19^+^ Nalm-6 cells for 6 hours at an E:T ratio of 1:1, assessed by CD107a expression. (E) Cytolysis by mock-transduced T-cells and CAR T-cells (HD, n=3) after 18 hours of co-culture with CD19^−^ U937 cells, donor-matched autologous B-cells, CD19^+^ Mec-1 cells or CD19^+^ Nalm‑6 cells at an E:T ratio of 1:1, defined as (% Live Cells (Target cells alone) - % Live Cells (Sample of Interest)) / % Live Cells (Target cells alone) x 100. (F) Apoptosis of primary chronic lymphocytic leukemia (CLL) cells on BMSCs upon co-culture with mock-transduced T-cells or CAR T-cells (HD, n=2) determined by continuous live cell imaging for 36 hours. Apoptotic CLL cells are double positive for Cytolight green and Annexin red. (G) Representative proliferation histograms for Cytopainter Red-labelled mock-transduced T-cells and CAR T-cells (HD, n=3) after 4 days in monoculture (upper panels) or co-culture with donor-matched B-cells (lower panels). The center plot shows proliferating CAR T-cells compared to mock-transduced T-cells after 4 days of co-culture with donor-matched B-cells, determined by % Cytopainter CP^Dim^ gate, based on the mock-transduced T-cell monoculture as reference on day 4. Data are shown as individual values with indication of the mean. On the right, the expansion of CD3^+^ CAR^+^ T-cells is shown upon target cell engagement. Data were analyzed by Two-way ANOVA with Šidák´s multiple comparisons test (C-E) and two-tailed unpaired t-test (G). Data are presented as mean ± SEM. Significance is indicated by * p = 0.05, ** p = 0.01, *** p = 0.001, **** p = 0.0001, ns = not significant.


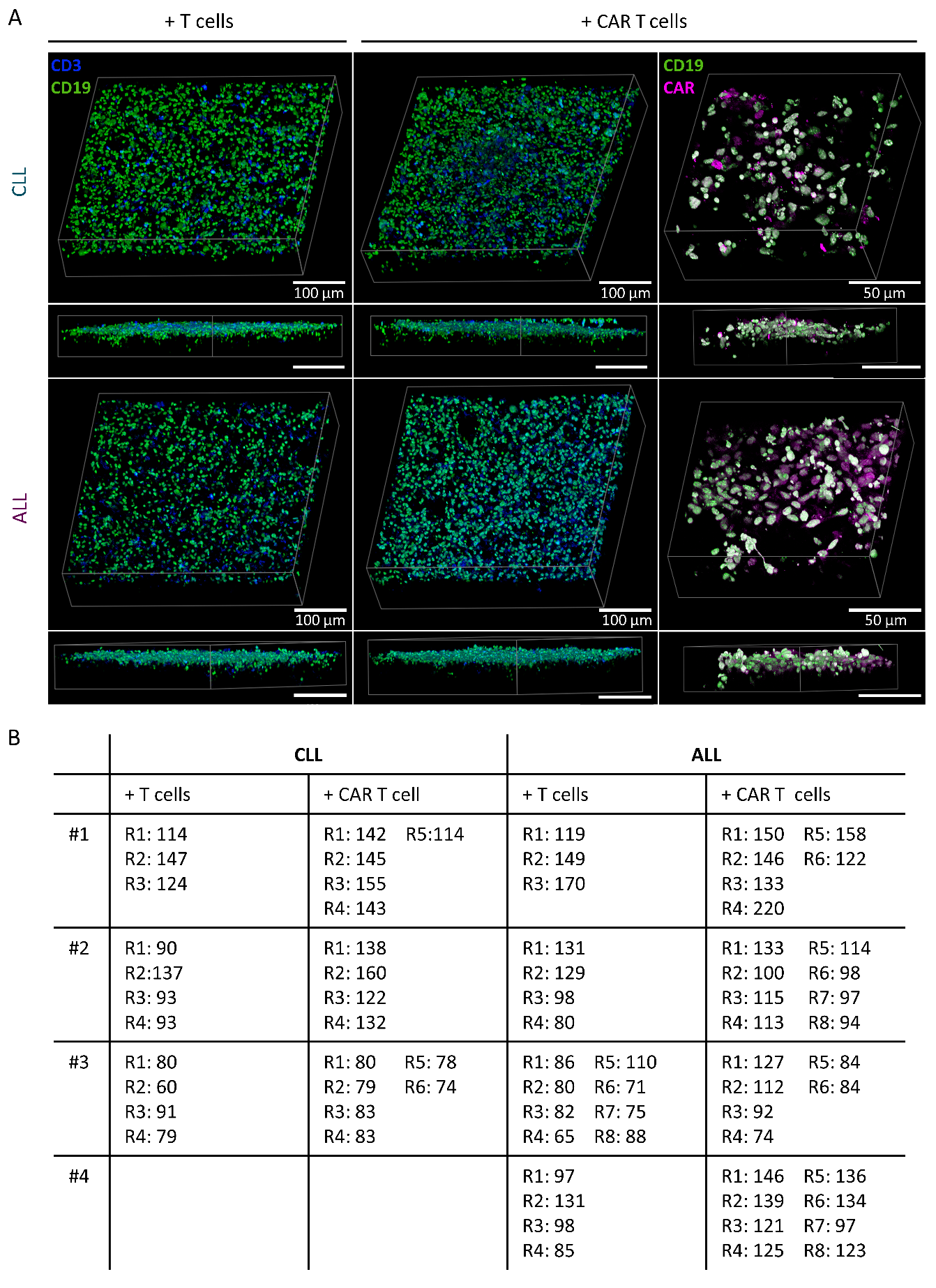


**Supplement Figure S6: 3D reconstructions of co-cultures with T-cells and malignant B cells within the 3D co-culture.** (A) Representative 3D reconstructions of co-cultures of CLL (upper panel) and ALL (lower panel) cells with autologous T-cells (left Panel) or CAR T-cells (right Panel) for 18 hours. The visualizations show the distribution of T-cells or CAR T-cells and malignant B cells within the scaffold-based 3D co-culture model. Z-stacks of the stained 3D co-cultures including B-cells (CD19, green) and T-cells (CD3, blue) were analyzed using Fiji software (Upper panel: number of stacks from left to right: 114, 114, 207; size of stacks: 1.04 µm, 1.04 µm, 0.4 µm; lower panel: number of stacks from left to right: 119, 158, 162; size of stacks: 1.04 µm, 1.04 µm, 0.34 µm). The higher magnification on the right highlights the co-localization of CAR T‑cells (CAR^+^, magenta) with malignant B-cells (magenta and green overlap). (B) Number of Z-stacks and Regions (R) analyzed for each patient.


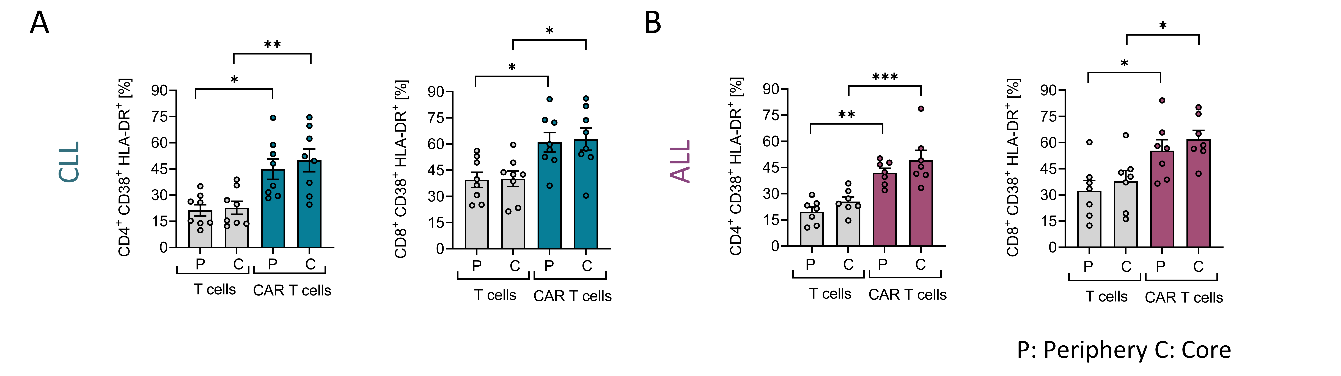


**Supplement Figure S7: Late activation of CLL and ALL patient-derived CAR T-cells.** Percentages of late activated CD38^+^ and HLA-DR^+^ (A) chronic lymphocytic leukemia (CLL, n = 8) or (B) acute lymphoblastic leukemia (ALL, n = 7) patient-derived mock-transduced T-cells and CAR T-cells after 4 days of co-culture.


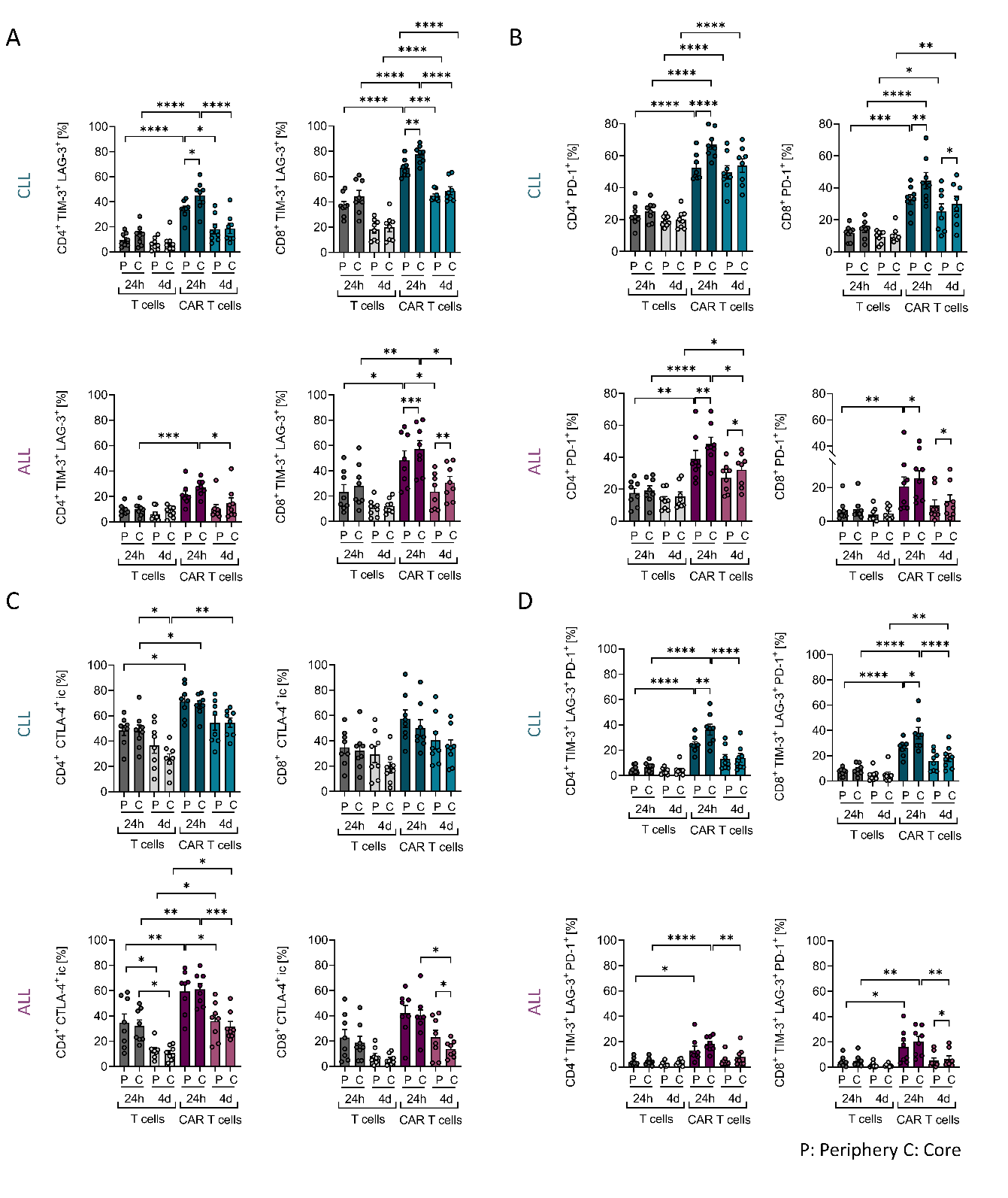


**Supplement Figure S8: Exhaustion of CLL and ALL patient-derived mock-transduced T-cells and CAR T-cells in 3D co-culture.** Percentages of exhausted (A) TIM-3^+^ and LAG-3^+^, (B) PD-1^+^, (C) intracellular CTLA-4^+^ and (D) terminal exhausted triple-positive TIM-3^+^ LAG-3^+^ PD-1^+^ CD4^+^ and CD8^+^ chronic lymphocytic leukemia (CLL) and acute lymphoblastic leukemia (ALL) patient-derived mock-transduced T-cells and CAR T-cells after 24 hours and 4 days of autologous 3D co-culture (n=8). Data were analyzed by Tukey’s multiple comparisons test and paired t-test for comparisons between peripheral and core regions. Comparison between ALL- and CLL-derived CAR T-cells was performed by Tukey’s multiple comparisons test (Significant differences for D: 24 h: CD4⁺ C −17.9 % ± 4.4; CD8⁺ C: −17.1 % ± 5.6; 4 days CD8^+^ −10.6 % ± 3.7; C −11.9 % ± 3.7) Bars represent mean ± SEM. Significance is indicated by *p = 0.05, **p = 0.01, ***p = 0.001, ****p = 0.0001. P: Periphery; C: Core.

**
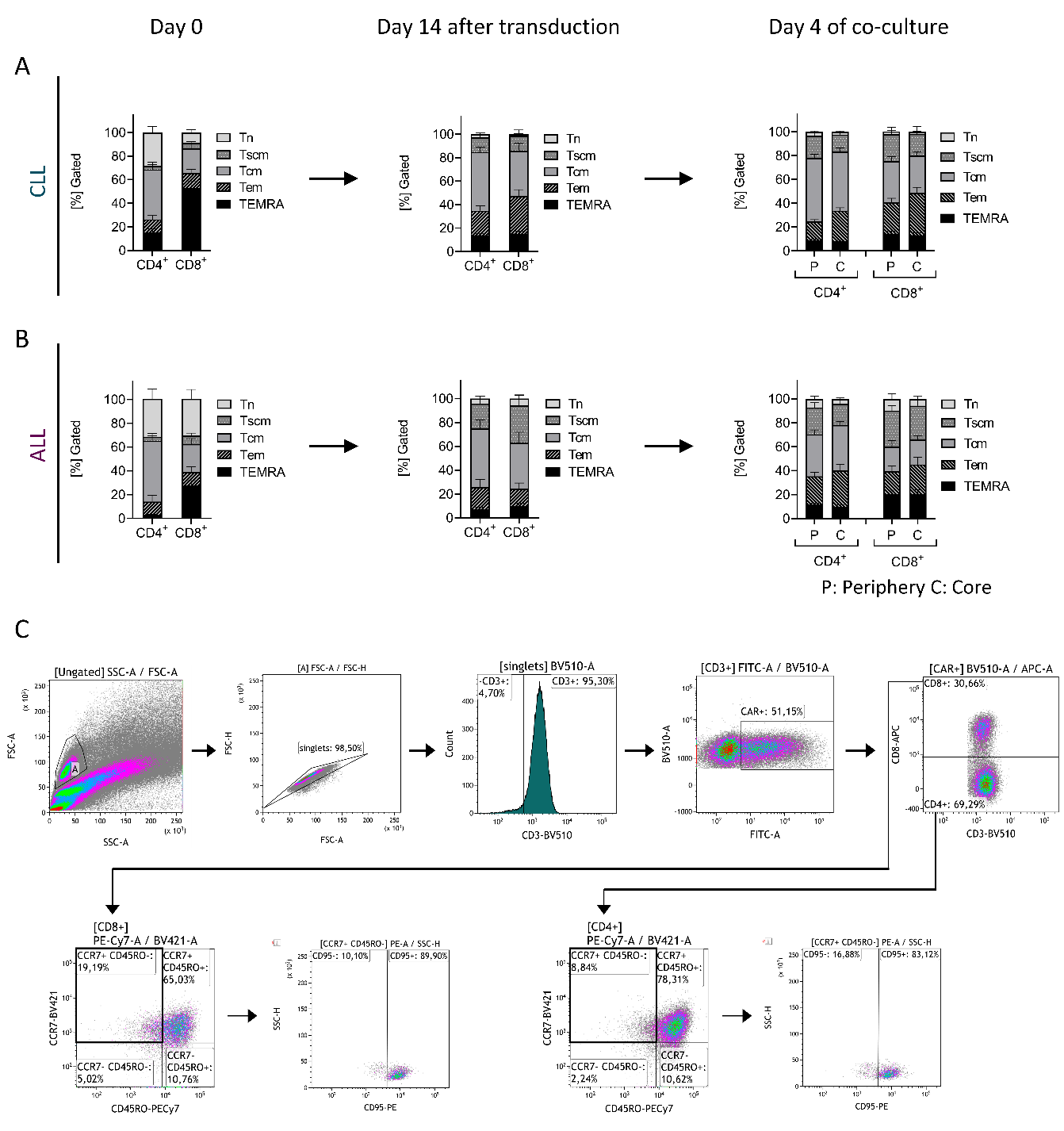
**

**Supplement Figure S9: Differentiation status of CLL and ALL patient-derived T-cells before and after transduction and following 3D co-culture.** Fractions of memory phenotypes of enriched CD4^+^ and CD8^+^ T-cells from (A) chronic lymphocytic leukemia (CLL, n=8) and (B) acute lymphoblastic leukemia (ALL, n=8) patient-derived PBMCs on day 0 before transduction and of mock-transduced T-cells on day 14 after transduction and day 4 of co-culture. (B) Representative gating strategy of flow cytometric analysis of CAR T-cells for CCR7, CD45RO and CD95 distinguishing naïve T-cells (Tn, CCR7^+^/CD45RO^-^/CD95^‑^), stem cell-like T-cells (Tscm, CCR7^+^/CD45RO^-^/CD95^+^), central memory T-cells (Tcm, CCR7^+^/CD45RO^+^), effector memory T-cells (Tem, CCR7^-^/CD45RO^+^) and Tem re-expressing CD45RA (TEMRA, CCR7^-^/CD45RO^-^). Cell debris and stromal cells of the co-culture were excluded by FSC-A/SSC-A, doublets were excluded by FSC-H/FSC-A and CD3^+^ T-cells further subdivided into CAR^+^ T-cells. CAR^+^ cells were subdivided into CD4 and CD8 T-cells and analyzed for CD45RO, CCR7 expression. CCR7^+^/CD45RO^-^ cells were further subdivided into CD95^+^ Tscm and CD95^-^ Tn cells.


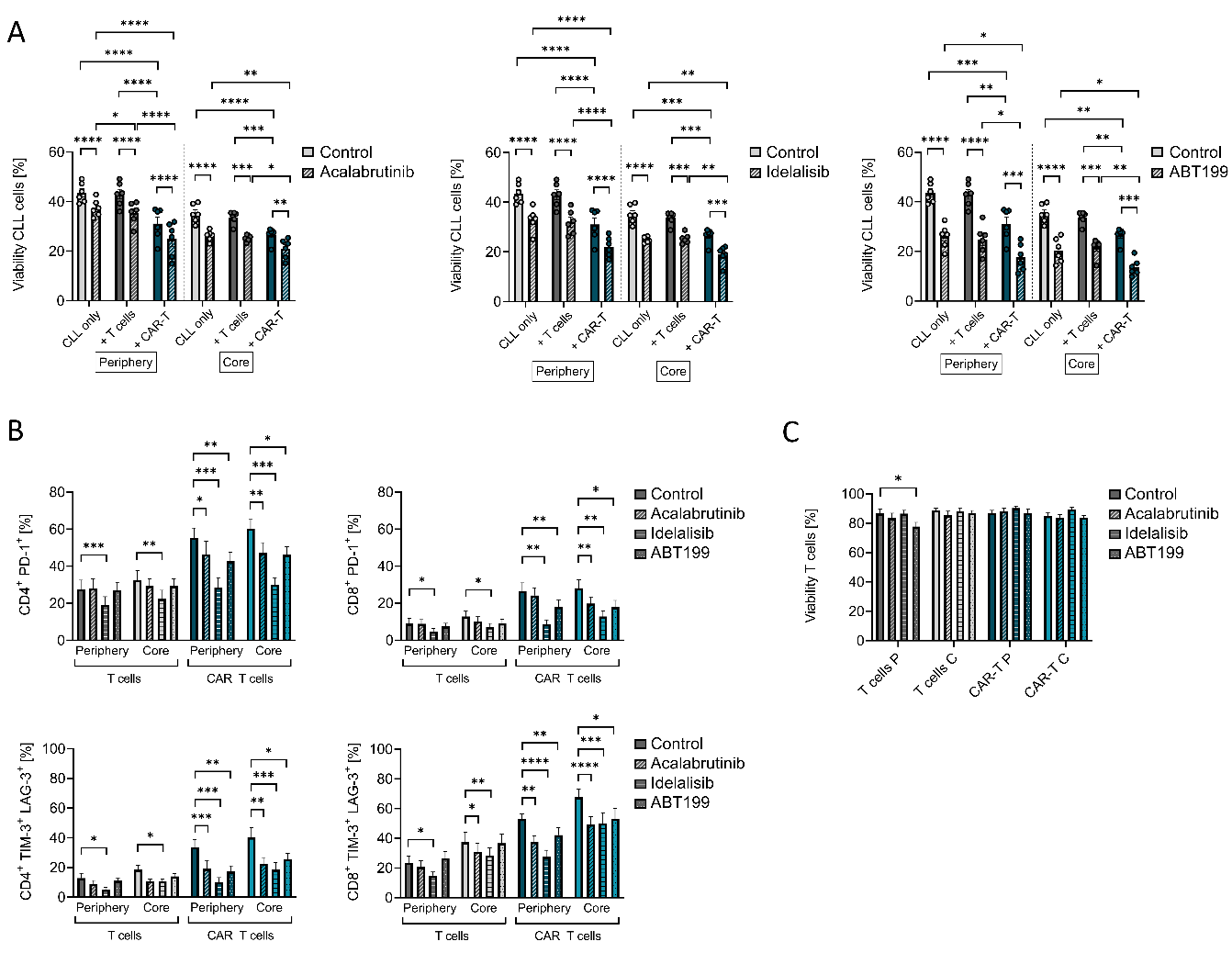


**Supplement Figure S10: Treatment of autologous CLL-derived 3D co-cultures with CAR T cells in combination with clinically approved inhibitors.** (A) Viability of chronic lymphocytic leukemia (CLL) cells after treatment of CLL co-cultures (n=6) with Acalabrutinib (10 µM), Idelalisib (5 µM) or ABT199 (10nm) for 72 h hours. After 48 hours of inhibitor treatment mock-transduced T-cells or CAR T-cells were added. CLL co-cultures without effector cells were used as control. 0.1% DMSO was used as solvent control. Data were analyzed by Tukey`s multiple comparison (Two-way ANOVA). (B) Percentages of exhausted CD4^+^ and CD8^+^ PD-1+ (upper panel) and TIM-3^+^ LAG-3^+^ (lower panel) mock-transduced T-cells and CAR T-cells after treatment of autologous CLL co-cultures with Acalabrutinib, Idelalisib or ABT199 as described in (A). Data were analyzed by paired t-test. (C) Viability of CLL-derived T-cells and CAR T-cells after treatment with Acalabrutinib, Idelalisib or ABT199 assessed by Annexin V/7-AAD staining (n=6). Data were analyzed by Dunnett’s multiple comparison test (Two-way ANOVA). Bars represent mean ± SEM. Significance is indicated by ns = not significant, *p = 0.05, **p = 0.01, ***p = 0.001, ****p = 0.0001. P: Periphery; C: Core.

**Supplement Table S1:** List of CLL patient characteristics.

| **Case no.** | **Gender** | **Age** | **Number of previous treatments** |
| --- | --- | --- | --- |
| #2008 | f | 82 | 0 |
| #0708 | m | 55 | 0 |
| #0703 | f | 59 | 0 |
| #0211 | m | 83 | 1 |
| #2005 | m | 71 | 0 |
| #3009 | f | 67 | 0 |
| #0795 | f | 83 | 1 |
| #1104 | f | 82 | 1 |
| #1405 | m | 81 | 0 |
| #0904 | f | 71 | 0 |
| #1610 | f | 68 | 1 |
| #1809 | f | 76 | 1 |
| #2401 | m | 55 | 0 |

m: male; f: female.

**Supplement Table S2:** List of ALL patient characteristics.

| **Case no.** | **Gender** | **Age** | **Subtype** | **Number of previous treatments** |
| --- | --- | --- | --- | --- |
| #0860 | m | 21 | Common-B (II) | First diagnosis |
| #1161 | f | 73 | Pre-B (B-III) | First diagnosis |
| #1163 | m | 21 | Pro-B (B-I) | First diagnosis |
| #0879 | f | 43 | Common-B (II) | First diagnosis |
| #1078 | m | 58 | Pre-B (B-III) | First diagnosis |
| #1197 | m | 81 | B-ALL biphenotypic | First diagnosis |
| #005 | n.a. | 15 | n.a. | n.a. |
| #007 | n.a. | 64 | n.a. | n.a. |
| #012 | n.a. | 38 | n.a. | n.a. |

m: male; f: female; n.a.: not available.

**Supplement Table S3:** List of antibodies and staining reagents.

| **Antibody/Reagent** | **Fluorochrome** | **Clone** | **Species/Isotype** | **Company** |
| --- | --- | --- | --- | --- |
| 7-AAD | - | - | - | BD Biosciences |
| Annexin V | FITC | - | - | Biolegend |
| CCR7 | BV421 | 150503 | Mouse IgG2a | BD Biosciences |
| CD3 | FITC | SK7 | Mouse IgG1, κ | BD Biosciences |
| CD3 | BV510 | UCHT1 | Mouse IgG1, κ | Biolegend |
| CD4 | PerCP | OKT4 | Mouse IgG2b, κ | Biolegend |
| CD4 | APC | SK3 | Mouse IgG1, κ | BD Biosciences |
| CD4 | BV421 | RPA-T4 | Mouse IgG1, κ | BD Biosciences |
| CD4 | PE-Cy7 | SK3 | Mouse IgG1, κ | BD Biosciences |
| CD8 | PE-Cy7 | SK1 | Mouse IgG1, κ | BD Biosciences |
| CD8 | BV421 | RPA-T8 | Mouse IgG1, κ | BD Biosciences |
| CD8 | FITC | SK1 | Mouse IgG1, κ | BD Biosciences |
| CD19 | BV421 | HIB19 | Mouse IgG1, κ | Biolegend |
| CD19 CAR FMC63 | PE | REA1297 | human IgG1 | Miltenyi |
| CD19 CAR FMC63 | VioBright515 | REA1297 | human IgG1 | Miltenyi |
| CD38 | PE-Cy7 | HB7 | Mouse IgG1, κ | BD Biosciences |
| CD45RO | PE-Cy7 | UCHL-1 | Mouse IgG2a, κ | BD Biosciences |
| CD69 | PE | CH/4 | Mouse IgG2a, κ | Invitrogen |
| CD95 | PE | DX2 | Mouse IgG1, κ | BD Biosciences |
| CD107a | BV421 | H4A3 | Mouse IgG1, κ | Biolegend |
| CD137 | APC | 4B4-1 | Mouse IgG1, κ | BD Biosciences |
| CXCL12 | AZDye 647 | - | - | Biolegend |
| CXCR4 | PE-Cy7 | 12G5 | Mouse IgG2a, κ | Biolegend |
| CTLA-4 | PE | BNI3 | Mouse IgG2a, κ | BD Biosciences |
| HLA-DR | PE | L243 | Mouse IgG2a, κ | BD Biosciences |
| Ki67 | APC | Ki-67 | Mouse IgG1, κ | Biolegend |
| LAG-3 | PE-Cy7 | 11C3C65 | Mouse IgG1, κ | Biolegend |
| PD-1 | BV421 | EH12.2H7 | Mouse IgG1, κ | Biolegend |
| TIM-3 | PE | F38-2E2 | Mouse IgG1, κ | Biolegend |
| Whitlow/218 Linker | AF 488 | E3U7Q | Rabbit IgG | Cell Signaling Technology |
| Whitlow/218 Linker | PE | E3U7Q | Rabbit IgG | Cell Signaling Technology |
| CD3 | - | F7.2.38 | Mouse IgG1, κ | Invitrogen |
| CD19 | - | 6OM P31 | Rat IgG2a, κ | Invitrogen |
| CD90 | - | JF10-09 | Rabbit IgG | Invitrogen |
| Anti-mouse | AF 405 | - | Goat IgG | Invitrogen |
| Anti-rabbit | AF 647 | - | Goat IgG | Invitrogen |
| Anti-rat | AF 594 | - | Goat IgG | Invitrogen |
| Mouse IgG1, κ | PE-Cy7 | MOPC-21 | Mouse IgG1, κ | BD Biosciences |
| Mouse IgG1, κ | FITC | X40 | Mouse IgG1, κ | BD Biosciences |
| Mouse IgG1, κ | APC | X40 | Mouse IgG1, κ | BD Biosciences |
| Mouse IgG1, κ | PE | X40 | Mouse IgG1, κ | BD Biosciences |
| Mouse IgG1, κ | BV421 | MOPC-21 | Mouse IgG1, κ | Biolegend |
| Mouse IgG2a, κ | PE-Cy7 | MOPC-173 | Mouse IgG2a, κ | BD Biosciences |
| Mouse IgG2a, κ | PE | X39 | Mouse IgG2a, κ | BD Biosciences |
| Mouse IgG2a, κ | BV421 | G155-178 | Mouse IgG2a, κ | BD Biosciences |
